# Distinguishing extant elephants ivory from mammoth ivory using a short sequence of cytochrome b gene

**DOI:** 10.1038/s41598-019-55094-x

**Published:** 2019-12-11

**Authors:** Jacob Njaramba Ngatia, Tian Ming Lan, Yue Ma, Thi Dao Dinh, Zhen Wang, Thomas D. Dahmer, Yan Chun Xu

**Affiliations:** 1 College of Wildlife and Protected Areas, Northeast Forestry University, Harbin, 150040 China; 2 BGI - Shenzhen, Shenzhen, 518083 China; 3 Laboratory of Genomics and Molecular Biomedicine, Department of Biology, University of Copenhagen, Copenhagen, DK-2100 Denmark; 4 China National GeneBank, BGI - Shenzhen, Shenzhen, 518083 China; 5 State Forestry and Grassland Administration Detecting Center of Wildlife, Harbin, 150040 China; 6 Ecosystems Ltd, No. 40 Shek Pai Wan Road, Aberdeen, Hong Kong China; 7 State Forestry and Grassland Administration Research Center of Engineering Technology for Wildlife Conservation and Utilization of China, Harbin, 150040 China

**Keywords:** Genetic markers, Genetic variation, PCR-based techniques

## Abstract

Trade in ivory from extant elephant species namely Asian elephant (*Elephas maximus*), African savanna elephant (*Loxodonta africana*) and African forest elephant (*Loxodonta cyclotis*) is regulated internationally, while the trade in ivory from extinct species of Elephantidae, including woolly mammoth, is unregulated. This distinction creates opportunity for laundering and trading elephant ivory as mammoth ivory. The existing morphological and molecular genetics methods do not reliably distinguish the source of ivory items that lack clear identification characteristics or for which the quality of extracted DNA cannot support amplification of large gene fragments. We present a PCR-sequencing method based on 116 bp target sequence of the cytochrome *b* gene to specifically amplify elephantid DNA while simultaneously excluding non-elephantid species and ivory substitutes, and while avoiding contamination by human DNA. The partial Cytochrome *b* gene sequence enabled accurate association of ivory samples with their species of origin for all three extant elephants and from mammoth. The detection limit of the PCR system was as low as 10 copy numbers of target DNA. The amplification and sequencing success reached 96.7% for woolly mammoth ivory and 100% for African savanna elephant and African forest elephant ivory. This is the first validated method for distinguishing elephant from mammoth ivory and it provides forensic support for investigation of ivory laundering cases.

## Introduction

Extant elephant species, *viz*. Asian elephant (*Elephas maximus*), African savanna elephant (*Loxodonta africana*), and African forest elephant (*Loxodonta cyclotis*) have suffered severe population declines due to habitat loss arising from rapid expansion of human settlement in their former ranges, poaching, and illegal ivory trade^1,2^. The conservation status of the two African elephant species was assessed by IUCN and listed in its Red List of Threatened Species as globally Vulnerable (VU) to extinction. Both species are assessed under *L. africana* because *L. cyclotis* was formerly considered to be conspecific with *L. africana*. Asian elephant was assessed by IUCN as globally Endangered (EN) in light of its declining global population^3^. In order to counter the threats of poaching, trading and utilization, international trade in African elephant specimens, including ivory, is prohibited by the Convention on International Trade in Endangered Species of Wild Flora and Fauna (CITES), with the exception of a few populations in Africa^4^. Meanwhile, domestic commercial trade of ivory is also strictly regulated and even banned in elephant range countries and consuming countries^5,6^. However, such strategies intended to break the supply chain often have not changed the social and cultural factors driving ivory trade or reduced demand^7^. There are two ways in which illegal dealers illegally trade in ivory yet avoid legal sanctions: one is to make crafts appear old and trade them as antiques, the other is to sell crafts in the name of other legal materials^8^. Woolly mammoth (*Mammuthus primigenius*) is an extinct species of the Elephantidae^9–11^ yet unearthed carcasses often retain high quality, carvable ivory of quality similar to that of the ivory of modern elephants^12^. Because mammoth ivory is a fossil and unrenewable resource, neither CITES nor national trade bans are applicable to it^13^. This loophole in ivory trade bans enables traders to fraudulently market elephant-derived ivory and crafts as products derived from fossil mammoths^5,14^.

Separation of mammoth ivory from extant elephant ivory has traditionally been based on a morphological method that examines Schreger lines and angles^15,16^. However, Schreger lines are not always clear on all ivory specimens. They are often undetectable when the ivory is highly processed, especially on products derived from the central section of the tusk. As alternatives, DNA based methods of species identification have been developed^17–19^. All these methods use mitochondrial DNA markers, i.e., the genes encoding partial Cytochrome *b* (Cyt *b*) and NADH-ubiquinone oxidoreductase chain 5 (ND5) but through different analytical strategies. Lee, *et al*.^20^ used a nested PCR and amplicon sequencing approach to obtain Cyt *b* fragments ranging from 188 bp to 402 bp. Assessment based on genetic distance demonstrated the validity of this method and its accuracy. However, the fragment sizes required by this method are too large to be amplified from DNA templates recovered from mammoth ivory because such DNA could have been highly degraded and deaminized during the fossilization process^21^. A recent study designed highly sensitive Mini-SNaPshot multiplex assays to obtain up to 233 bp fragments of Cyt-*b* gene from ivory samples although the only mammoth sample that was examined failed to amplify and the outcome on mammoth samples could not be confirmed^19^. Given that woolly mammoth DNA fragments longer than 180 bp have consistently failed to be amplified^22^, analysis of shorter fragments (<180 bp) containing sufficient informational content to permit distinction of elephantid species could be suitable. Wozney and Wilson^18^, and Kitpipit *et al*.^19^ used smaller target fragments of Cyt *b*^18^ and ND5^19^ genes that are better suited to analyses of shorter fragments including mammoth ivory DNA. However, assays proposed in these studies may have possible mismatches for certain elephantid mtDNA haplotypes considering that some elephantid sequences in GenBank (e.g. Accession No. AY769974, AY769975, AY769973, DQ316068 when aligned with Wozney and Wilson^18^ assays) display multiple mismatches on the critical five last nucleotides at the 3′ end of the primer binding region, which may dramatically affect amplification efficiency or even cause amplification failure^23,24^. Thus, it is difficult to tell whether these methods are to effectively analyse elephantid samples derived from such mtDNA haplotypes. Additionally, the identification approach used in these methods was solely based on species-specific nucleotides, a basis that increases risk of misidentification if intra-specific variation is not well represented among reference samples. Given that the number of woolly mammoth samples used in these studies was only 1 or 2, it is difficult to conclude that these methods are to effectively distinguish mammoth ivory from elephant ivory in forensic practice.

In addition to elephantid tusk, the teeth of other species are commonly used as ivory substitutes (especially in carving and crafting), including common hippopotamus (*Hippopotamus amphibius*), common warthog (*Phacochoerus aethiopicus*), narwhal (*Monodon monoceros*), sperm whale (*Physeter catodon*), killer whale (*Orcinus orca*), and walrus (*Odobenus rosmarus*)^25^. Ivory is also often substituted with cattle bone^26^. In practice, forensic cases involving evidence suspected to be elephantid ivory often needs to exclude other alternative ivory or substitutes. To our knowledge, only morphological methods are available at present^25^. It is difficult to satisfy the identification needs for carvings on which diagnostic characteristics cannot be detected.

Another issue for ivory DNA analysis is human contamination because ivory specimens are repeatedly handled by humans from the time they are obtained to processing and trading. Contaminating human DNA can act as template during PCR with priority over scarce and highly degraded DNA recovered from fossilized mammoth ivory^27^, and this can lead to spurious results. To avoid this outcome, practitioners are advised to use great caution during DNA isolation^28^.

Considering these requirements for molecular species identification of elephantid ivory and their products, methods that are most applicable should have: (1) high resolution to separate extant and fossil elephantid ivory and exclude products from other species that are used as alternative ivory or substitutes; (2) guaranteed experimental success rate; and (3) ability to avoid human DNA contamination. Our study objective was to test whether amplification of a fragment of the Cyt *b* gene would yield results that would meet these three criteria. The currently proposed method is preferred as a means of avoiding contamination, excluding non-elephantid species, amplifying amplicons that are short enough yet informative to discriminate elephantids, and improving inclusivity, thus extending the spectrum of elephantid species identification.

## Results

### Reproducibility and sensitivity

Primer pair L15123/H15240 successfully amplified PCR products in 100% of Asian elephant samples (61/61), 96.7% of woolly mammoth (59/61), 100% (22/22) of African savanna elephant and 100% (8/8) of African forest elephant, but 0% of human (0/5), hippopotamus (0/4), white rhino (0/6) and domestic cattle samples (0/4) (Supplementary Table S1). All PCR amplicons (160 bp, with flanking primer sequences incorporated) were successfully sequenced and the primer sequences subsequently trimmed to obtain actual gene fragment sequences of 116 bp, which confirmed their correctness for all elephantid species with percent nucleotide similarity (%NS) of 100% (except for one sample that showed 99.22% similarity) for Asian elephant, and 100% for African savanna elephant, African forest elephant and woolly mammoth (Supplementary Table S2).

Primer blast in GenBank using the NCBI Primer-Blast software showed the top 250 hits included 117 *Mammuthus primigenius*, 31 unspecified *Mammuthus* species (*Mammuthus sp*.), 19 *Loxodonta cyclotis*, 2 *Elephas maximus*, 62 *Loxodonta africana*, 2 *Mammuthus jeffersonii* and 17 *Mammuthus columbi*. Although the top 250 hits are highly dependent on the number of GenBank database sequences represented by closely matching species, 80% of the total top 250 hits matched the target elephantid species sequences (i.e. 46.8% *M. primigenius*, 7.6% *L. cyclotis*, 0.8% *E. maximus* and 24.8% *L. africana* halpotypes) in the database, which could vary upwards depending on the true identity of the sequence hit matches identified as *Mammuthus sp*. These *in silico* tests showed a high degree of specificity to the target elephantid species and provided an indication of other potential mammoth species the primers might amplify. All non-target mammoth species sequences (*Mammuthus jeffersonii, Mammuthus columbi* and *Mammuthus sp*.) identified in this test showed 100% nucleotide match with primer sequences and similar annealing stabilities. Thus, we concluded it was highly likely that our designed primer pair could also amplify these two ancient elephantid species.

**Figure 1:**
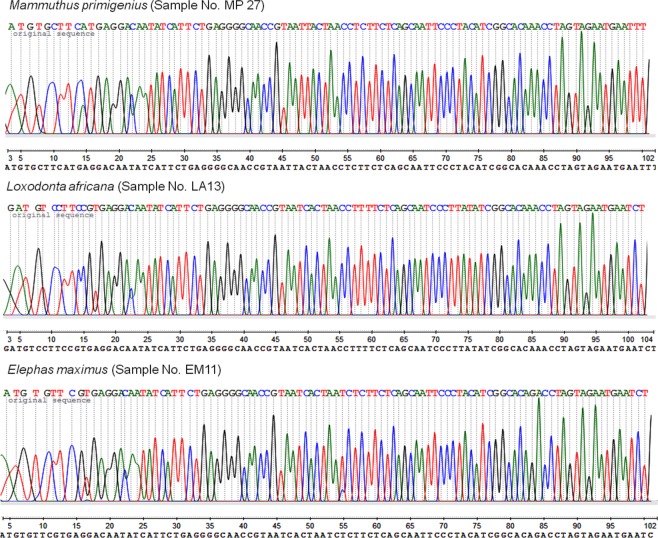
Sequencing chromatogram of amplicons of mixed DNA samples. Mammoth (*M. premigenius*), African savanna elephant (*L. africana*) and Asian elephant (*E. maximus*) sequences obtained from PCR using DNA mix of mammoth-human, African savanna elephant-cattle and Asian elephant-cattle in 1:5 elephantids to non-elephantids concentration ratio.

The mixed DNA tests showed that the mammoth-human DNA mix, the African savanna elephant-cattle mix, and the Asian elephant-cattle mix all generated a single PCR product at the expected fragment size. All sequencing chromatograms of these amplicons were clean and showing neither significant overlapping signals (peaks) nor strong noise signals (Fig. 1). Sequence analysis showed that these amplicons from mixed DNA samples were all derived from mammoth and elephant with no exception, demonstrating the high elephantid specificity of the primer pair.

### Resolution for species identification

For the 116 bp target fragment of the four species of elephantids, alignment of sequences generated from this study and those in GenBank revealed a total of 16 variable (polymorphic) nucleotides within this fragment, for which 8 of them were observed among interspecies. Among the 8 variable nucleotides, 4 were found to be specific to the S clade savanna elephants. The level of intraspecific nucleotide variation ranged widely. Nucleotides variations within species was, 4 for savanna elephant (1 for S clade and 3 for F clade), 4 for forest elephant (F clade), 1 for woolly mammoth, and 0 for Asian elephant (Table 1). It should be stressed that, savanna elephant sequences generated from this study and used in subsequent analyses were found to belong to S clade. Meanwhile, the calculated nucleotide diversity (π) of the four elephantid species was 0.0338, while the π for each species was 0.0049, 0.0059, 0.0027 and 0 for savanna elephant, forest elephant, woolly mammoth and Asian elephant respectively. Nucleotide diversity levels for each species were much smaller than the overall level.

**Table 1:** The positions of the interspecific polymorphic nucleotides (in bold) on the 116 bp cytochrome *b* sequence region of the four elephantid species, and the position of the intraspecific polymorphic nucleotides.

Nucleotide polymorphism															
***Species***		15124	**15148**	15151	15160	**15184**	15190	**15193**	**15205**	**15208**	15210	**15211**	**15221**	15222	**15238**
*L.cyclotis*	F clade	T/C	G	G/A	A/G	C	C	C	T	C	A	C	A	G/A	C
*L.africana*	F clade	C	G	A/G	A/G	C	C	C	T	C	A	C	A	A/G	C
	S clade	C	G	A	A	C	C	T	C	T	A/G	T	A	A	C
*M. primigenius*		C	G	A	A	C	C/T	C	T	C	A	C	G	A	C
*E.maximus*		C	A	A	A	T	C	C	T	C	A	C	A	A	T

Nucleotide position numbers are in accordance with the revised Cambridge Reference Sequence (rCRS) for the human. Owing to historical hybridisation and introgression events, the F clade savanna elephants have been found to carry only the mtDNA of forest elephant. Nucleotide variations in African elephant species (*L. africana* and *L.cyclotis*) are therefore displayed as F clade (present in both African elephant species) and S clade (present in only *L. africana*).

In distance matrix estimation models of K2P and *p*-distance using Ts, the total number of intraspecific comparisons was 3863 pairs and for interspecific comparisons 8227 pairs. For K2P distance matrices, intra-*d*s ranged from 0 to 0.89% and averaged 0.14 ± 0.32%; inter-*d*s ranged from 1.80% to 7.49% and averaged 4.41 ± 1.38%. For *p*-distance matrices, intra-*d*s ranged from 0 to 0.87% and averaged 0.14 ± 0.32%; the inter-*d*s ranged from 1.77% to 6.96% and averaged 4.21 ± 1.25%. The inter-*d* matrix demonstrated sufficient resolution between the four elephantid species at this Cyt *b* region to conduct taxon identification (Table 2). The intra-*d* matrix demonstrated significantly less variation. The gap between the upper limit of intra-*d* and lower limit of inter-*d* at 95% confidence was 0.91% (0.89% to 1.80%) for K2P and 0.90% (0.87% to 1.77%) for *p*-distance, respectively.

**Table 2:** Matrices comprising the range of pairwise inter-*ds* (as percentage) for (116 bp) sequences from the four elephantid species, and the range of intra-*ds* (on the diagonal, bold).

Species	K2P-distances				p-distances			
	(1)	(2)	(3)	(4)	(1)	(2)	(3)	(4)
(1) *M. primigenius*	**0–0.89**				**0–0.87**			
(2) *L.cyclotis*	1.80–3.61	**0–0.89**			1.77–3.48	**0–0.87**		
(3) *L.africana*	4.55–6.49	4.55**–**6.49	**0–0.89**		4.35–6.10	4.35–6.10	**0–0.87**	
(4) *E.maximus*	3.61–4.55	3.61**–**4.55	6.49**–**7.49	**0–0**	3.48–4.35	3.48–4.35	6.10–6.96	**0–0**

**Figure 2:**
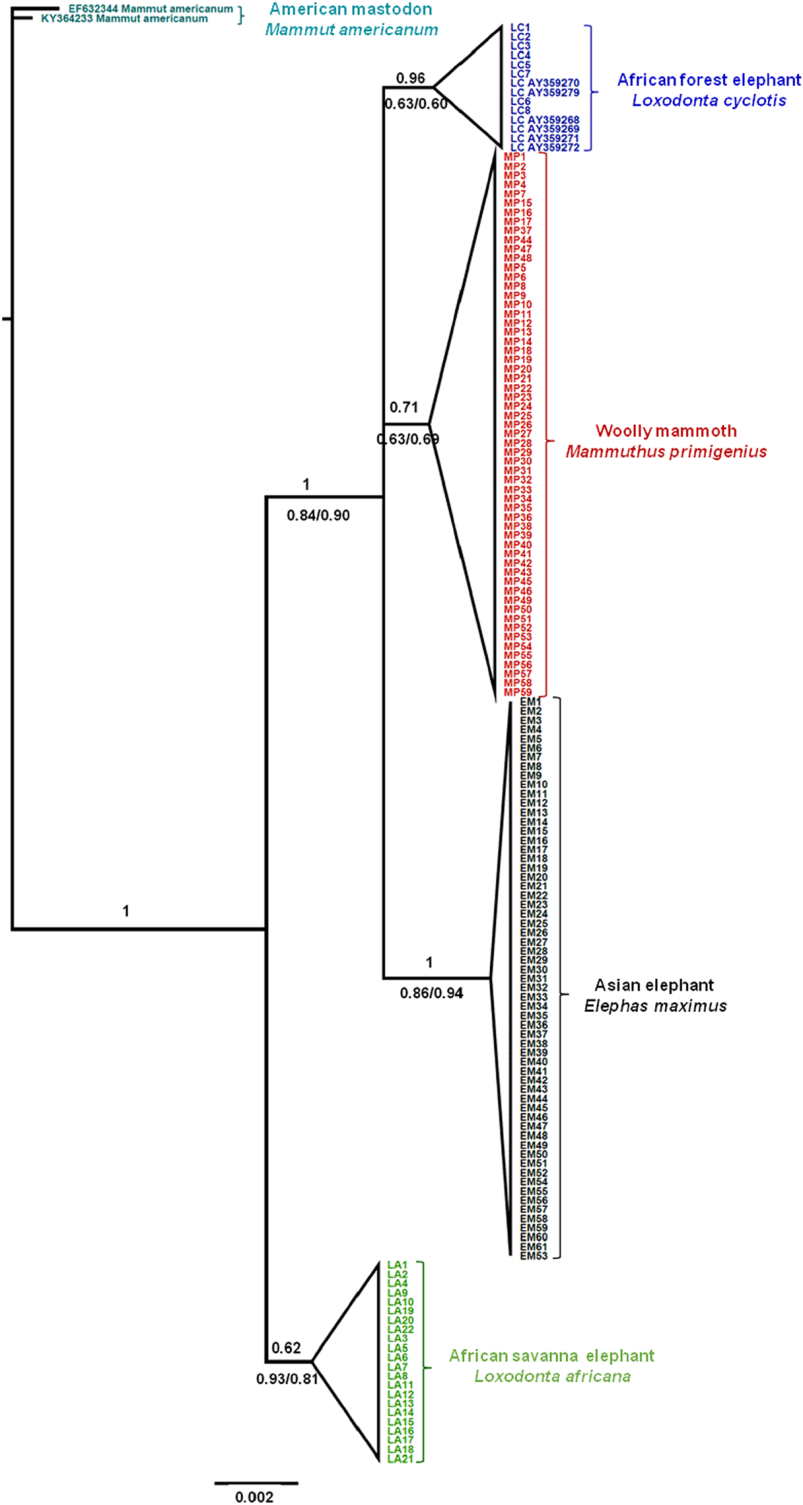
Bayesian phylogenetic analysis of the 116 bp Cyt *b* fragment amplified by primers L15123/ H15240. The posterior probabilities are shown above the corresponding branches and the bootstrap support values for the maximum likelihood (ML) analysis/Neighbor Joining (NJ) analysis are shown below the corresponding branches. All elephantid species clustered into separate lineages with high posterior probabilities and bootstrap support. However, we would note that other studies have found that many savanna elephants (F clade) carry forest-derived mtDNA lineages.

Phylogenetic trees were reconstructed using Maximum likelihood (ML), Neighbour Joining (NJ) and Bayesian phylogenetic analysis methods based on 150 elephantid sequences obtained in this study and 6 sequences of African forest elephant museum specimens downloaded from GenBank. All trees successfully clustered the four elephantid species into separate lineages (Fig. 2). Some of the tree branches were well supported at the species level with Bayesian posterior probability (PP) of >95%, while there was strong support (PP > 95%) at the nodal bases. ML and NJ estimations showed weak bootstrap support (BP) at both branches and species level (P < 95%). It should be noted that other studies have shown that, due to ancient hybridization events, some savanna elephants carry forest elephant-derived mtDNA^29–31^, although this was not evident among our samples (see Fig. 2), and would not affect the ability of our method to distinguish the African elephant (*Loxodonta*) genus from mammoths or Asian elephants. Two deeply divergent mtDNA clades have been detected in African elephants, namely, S clade (only found in savanna elephants) and F clade (found in all forest elephants and also in some savanna elephants)^29–31^. As such, studies using full-length mtDNA genes consistently group forest and savanna elephants together^30,32^. In the current study, Fig. 2 shows *Loxodonta africana* (S clade) as an outgroup to the three elephantid species, and this tree branching pattern is likely due to the limited length of sequences used, which does not allow resolution of true relationships. However, this does not affect the validity of the method or sequences as it is just a limitation of the short length of the sequences for inferring the true tree.

### Sensitivity

**Figure 3:**
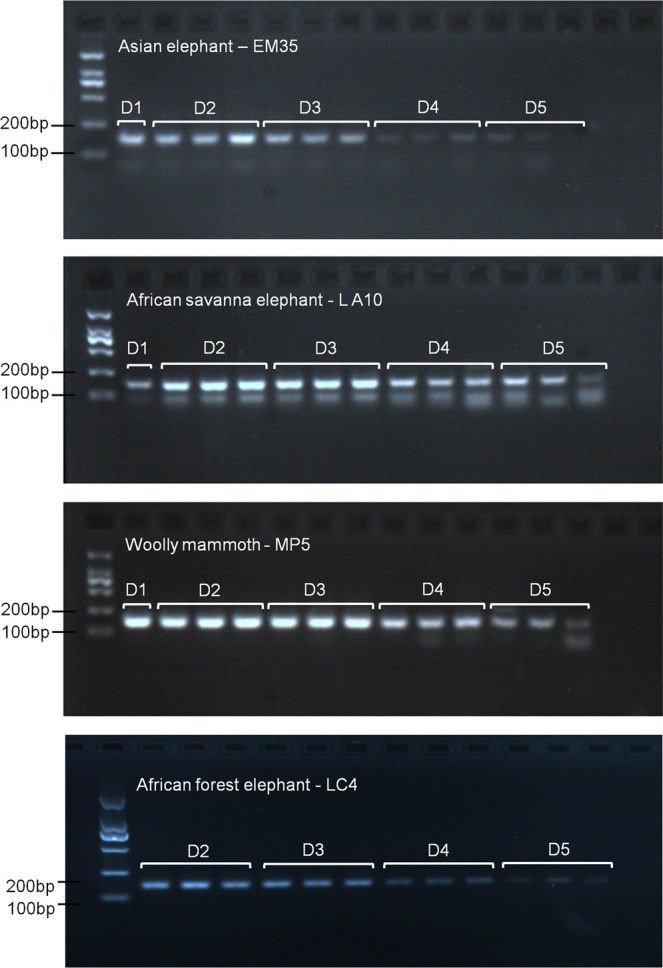
Agarose gel electropherogram of the sensitivity test. DNA extracted from an Asian elephant (#EM35), African savanna elephant (#LA10), and woolly mammoth (#MP5) was diluted in ~10 ng/µl (D1), ~1 ng/µl (D2), ~0.1 ng/µl (D3), ~10 pg/µl (D4) and 1 pg/µl (D5), while for African forest elephant (#LC4), four latter dilutions of DNA were done followed by amplification in triplicate through PCR using the L15123/ H15240 primers. The full length gels are presented in Supplementary Fig. S1.

Five serial quantities of DNA (~10 ng/µl, ~1 ng/µl, ~0.1 ng/µl, ~10 pg/µl and 1 pg/µl) from an Asian elephant (fecal DNA), an African savanna elephant (fecal DNA), and a woolly mammoth (ivory DNA) were used to test the detection limit of this PCR system. As mentioned in sensitivity test section below, only the four latter serial quantities of DNA from an African forest elephant (ivory DNA) were included in this test because the total quantity of isolated DNA was <10 ng/µl. As shown in Fig. 3, all four serial DNA dilutions were amplified at DNA input of 1 pg/µl, suggesting that the sensitivity of the PCR system was adequate to distinguish mammoth from African savanna elephant and African forest elephant ivory.

Considering the possibility that the input total DNA from feces and ivory might be contaminated, we tested the actual template copy numbers of the least input quantity of total DNA for which the target product is visible on agarose gel and available for sequencing. Absolute quantification of the ~10 ng/µl of input DNA from Asian elephant, savanna elephant, and woolly mammoth, demonstrated that the average copy numbers of this input quantity were 253896, 3670000 and 3066667 respectively. The sensitivity test of forest elephant ivory started from 1 ng/µl because the total quantity of isolated DNA was <10 ng/µl. The average copy numbers quantified for this sample were 10076. The detectable copy numbers in 1 pg/µl of input DNA were 25 copies for Asian elephant, 367 copies for woolly mammoth, 307 copies for savanna elephant and 10 copies for forest elephant. These results suggest that the developed assay can yield enough amplicons for sequencing from as low as 10 copies of the template DNA from extant ivory and 367 copies from fossilized ivory, demonstrating its potential effectiveness at identifying poor quality mammoth and elephant ivory samples.

## Discussion

Poaching and illegal ivory trade are prominent threats to the three extant elephant species^1,2^. Consistently accurate forensic identification of elephant ivory is essential to combat such crimes. It is increasingly urgent to distinguish extant ivory from mammoth ivory because unregulated trade in fossil ivory has become a cover for laundering elephant ivory^5,14^. This loophole will be sustained by increasing supply of woolly mammoth ivory from permafrost environments^33^. Here, we present an effective method based on molecular genetics to distinguish extant and fossil elephantid ivory when routine morphological methods cannot reach a reliable identification due to the absence of effective morphological characteristics on ivory samples.

We set three goals for designing the method: simultaneous identification of extant and fossil ivory, exclusion of substitute ivories, and avoidance of human DNA contamination. All these goals were simultaneously achieved by using the primers L15123/ H15240 on the Cyt *b* gene, which are universal to all elephantids but highly variable in nucleotide sequences in humans and among species whose teeth or bones are used as ivory substitutes. These primers were designed to amplify a DNA fragment of 160 bp (with flanking primer sequences incorporated) to improve the success rate for amplifying highly degraded and deaminized DNA recovered from fossil ivory^21^. Furthermore, the current method takes into account the mtDNA clade diversity of elephantid species (Supplementary Table S3) which demonstrates the potential utility of these primers for identification of a broad spectrum of elephantid mtDNA haplotypes. The primer set was validated for reproducibility, specificity and sensitivity following the recommendations on the use of animal (non-human) DNA in forensic investigation^34^.

The primer pair L15123/ H15240 had high success rate for different types of samples, 100% success for DNA isolated from dung samples (Asian elephant, n = 61, African savanna elephant, n = 20), 100% for extant elephant ivory samples (African forest elephant, n = 8, African savanna elephant, n = 2) and 96.7% success rate for woolly mammoth ivory samples (59/61). This demonstrates the reproducibility of these primers in species identification of elephantids. However, it’s worth noting that, although the primers showed a high success rate in almost all of our samples, there is always a potential risk of mismatches should any novel sequences have mutation, although this risk is quite low. The final target fragment, although as short as 116 bp, exhibited high polymorphism among the four elephantid species with an overall nucleotide diversity (π) of 0.0338. Intraspecific levels of π for all four species ranged from 0.0 to 0.0059, significantly lower than nucleotide diversity at taxonomic family level. This nature of intraspecific conservation and interspecific variation caters to the requirements for reliable species identification. Furthermore, both K2P pairwise genetic distance and *p*-distance showed a clear gap between intra- and-interspecific distances. All four species clustered into separate lineages in the Bayesian phylogenetic tree (Fig. 2) with strong support of branching at species level (Bayesian posterior probability >95%). These results show that the Cyt *b* gene fragment used in this study proved effective to distinguish the four elephantid species. In addition, *in silico* primer blast recorded 100% match with our primers L15123/ H15240 to the sequences of another two ancient mammoth species *M. columbi* and *M. jeffersonii*, demonstrating the potential for broader applicability to distinguish the ivory of these ancient mammoths from that of extant elephants.

However, mtDNA sequence-based method is not applicable to species assignment of hybrids, because mitochondrial genome inherits maternally and inherently identifies the matrilineal species of hybrids. The African forest and savanna elephants may hybridise in the wild and produce hybrids^35^. It has been observed that extensive introgression of mtDNA from the forest elephants into the savanna elephant’s populations^29^. As such, two deeply divergent clades of mtDNA have been detected in African elephants and are often referred as S clade (exclusively found in savanna elephant) or F clade (found in all forest elephants but also found in some savanna elephant populations that otherwise show no evidence of hybridisation)^29–31^. Our method can identify the S clade elephants as savanna elephants, but cannot assign F clade to forest elephant or hybrid species without examining the nuclear markers.

The primer pair L15123/H15240 was specifically designed to amplify DNA from elephantids. It contained 6 to 12 differential nucleotide sites to robustly exclude non-elephant species, including human. For specificity testing, we selected four species whose primer sequence region exhibited 6 to 12 nucleotide mismatches with the primer sequences as follows: hippopotamus (with 6 differential nucleotides, n = 4), white rhino (with 9 differential nucleotides, n = 4), domestic cattle (with 12 differential nucleotides, n = 4) and human (with 11 differential nucleotides, n = 6). We recorded 100% negative results for these species even when elephantid DNA was mixed with DNA of these species in 1:5 concentration ratio. For species providing ivory substitutes but not included in our test, such as warthog, narwhal, sperm whale, killer whale, and walrus^25^, all are phylogenetically distant from elephantids^36^, and have differential nucleotides in the primer sequence region varying from 6 to 12. Therefore, we are confident in concluding that they are not amplifiable by the primer pair L15123/H15240^37^.

DNA sources involved in forensic and conservation practice are often poor to provide high quality DNA^38,39^. Tolerance to DNA quality of a PCR-based method is thus essential for its applicability. By fully considering information content of target fragment, improving success rate is our main goal. Our validation tests for all extant species were performed using total DNA isolated from fresh faeces, frozen tissues and plucked hair follicles (Supplementary Table S1). In relation to the amplified fragment size of primers L15123/H15240, the quality and quantity of DNA from these samples were all adequate to generate products. However, the success rate for DNA recovered from extant and fossilized ivory is the real challenge of our method. It was reported that DNA isolated from extant elephant ivory could support amplification of fragments as long as 486 bp. When amplicons were reduced to 188 bp, overall success rate reached 84.3%^20^. In our tests, use of a 160 bp amplicon achieved 100% success rate for amplification of extant ivory (8 African forest elephant samples and 2 African savanna elephant samples). Meanwhile, mammoth ivory preserved underground for thousands years often contain degraded and deaminated DNA. Cytosine deamination is the most common form, which leads to the conversion of cytosine to uracil and often manifests as a base substitution C > T or G > A mutations on the DNA strand^40^. Cytosine deamination is most prevalent on the outermost few (~10) bases on the ends of DNA template, but occurs at low levels across the length of a degraded DNA molecule^41,42^. Such substitutions are not expected to affect the power of our assay to discriminate elephantids as the variable nucleotide observed among interspecies were largely found at the inner part of the target fragment. Our tests showed that amplification of mammoth ivory DNA achieved 96.7% success rate (59/61 samples), and completely separated mammoths from other species (Fig. 2). Nevertheless, as a precautionary measure, although not performed here, ivory DNA templates could be treated with uracil *N*-glycosylase (UNG) prior to PCR amplification, which could dramatically reduce potential cytosine deamination and the resultant mutations by over ~99.9%^40^.

The sensitivity test of a method often employs total DNA input expressed as nanogram (ng) or other mass units. This is straightforward to roughly estimate total DNA input for an experiment. In this study, we used same means to test the sensitivity of our PCR system and observed that this system could yield visible products on agarose gel when template input of elephantid DNA was as low as 1 pg/µl (Fig. 3). The limit of detection was lower compared to that used in previous reports^19,43^. Considering the low concentrations of DNA in elephant ivory (usually <1 ng/µl)^44^, and the low quantities of endogenous DNA in ancient specimens such as the woolly mammoths^45^, we further tested the minimum detectable copy number of template DNA. Results showed that our PCR system could detect as low as 10 copies of the template in a 20 µl system for extant ivory DNA and 367 copies for mammoth ivory DNA, suggesting wide applicability to ivory samples. However, where possible, and as a safeguard, larger DNA quantities should be used when testing forensic specimens^46^.

In summary, the method we developed allows species identification of ivory for both extinct woolly mammoths and extant elephant species. It was successfully validated and confirmed to be reproducible, accurate, and highly specific to elephantids. The short yet informative target fragment (116 bp) not only yielded good resolution for differentiating extant elephantid species from woolly (and possibly other mammoths), but also, by virtue of its easily amplifiable length, expanded applicability of the method to both extant and fossil ivories, the latter of which are often typically degraded. Meanwhile, the primer pair proved highly specific to elephantids and robustly excluded non-target species including human, thus avoiding human DNA contamination during forensic analysis. The application of this ivory identification system can improve the enforcement and prosecution of elephant ivory trafficking crimes, thereby serving as a deterrent to illegal trade in ivory^47^.

## Materials and Methods

### Sample collection and DNA extraction

Dung samples from African savanna elephant (n = 20), Asian elephant (n = 61), common hippopotamus (*Hippopotamus amphibius*) (n = 4) and white rhino (*Ceratotherium simum*) (n = 6) were collected within 12 hrs of defecation from 19 zoos across China (Supplementary Table S1). A razor blade was used to shave the surface of each dung sample, and the shavings were transferred to 100 ml bottles containing the DET buffer (20% DMSO, 100 mM Tris pH 7.5, 0.25 M EDTA, saturated with NaCl) in approximately 1:4 dung-to-solution ratio. These samples were stored at room temperature until or after DNA extraction. Fecal DNA extraction was performed using a QIAamp DNA Stool Mini Kit (Qiagen) with slight modifications as previously described^48^. Raw ivory samples of woolly mammoth (n = 61) were collected from ivory carving factories in China (Supplementary Table S1). Species of origin of each sample was confirmed based on Schreger angles^25^ prior to collection. All ivory samples were originally from the Sakha Republic (Yakutia) of the Russian Federation, the source of most mammoth ivory for carving and trading. Raw ivory samples of African savanna elephant (n = 2) and African forest elephant (n = 8) were donated by the State Forestry and Grassland Administration Detecting Center of Wildlife of China. DNA extraction from mammoth ivory samples was performed according to the established protocol for extracting ancient DNA from bones and teeth^49^, and DNA extraction of extant ivory samples was performed following the total demineralization method^50^. Four tissue samples of domestic cattle (*Bos taurus*) were donated by the State Forestry and Grassland Administration Detecting Center of Wildlife of China, and five sets of freshly plucked hair samples of humans (*Homo sapiens*) were donated by volunteers from our lab (Supplementary Table S1). The hair samples were first rinsed in deionised water before DNA extraction. DNA extraction from tissues and hair follicles, and purification of extracts was performed using AxyPrep Multisource Genomic DNA Miniprep Kit (AXYGEN, China) according to manufacturer instructions. All extracted DNA was quantified using NanoDrop Spectrophotometer (ND-1000, NanoDrop Technologies) and diluted to around 10 ng/μl unless the DNA concentrations of ivory samples were below this level.

### Primer design

A total of 52 complete Cyt *b* sequences (about 1137 bp) of African savanna elephant, African forest elephant, Asian elephant, woolly mammoth, common hippopotamus, warthog, narwhal, sperm whale, killer whale, walrus, domestic cattle, white rhinoceros and human were downloaded from GenBank (Supplementary Table S3). Sequences for elephantid species were chosen based on previously published mitochondrial DNA lineage (Clade) information and the completeness of the cytochrome *b* gene sequence (Supplementary Table S3). Sequences were aligned using MEGA 5.3^51^. The regions that are informative at both intra-specific and inter-specific levels and therefore suitable for designing elephantid - specific primers were screened. A 116 bp fragment was selected from the Cyt *b* region between nucleotide numbers 15123 and 15240 in accordance with the revised Cambridge Reference Sequence (rCRS) for the human mitochondrial genome^52,53^, and a pair of universal primers for elephantids was designed using Primer PREMIER 5 software (Premier Biosoft International, Palo Alto, CA, USA). The forward primer was L15123: 5′- TACTACTRATYACYATAGCCAC−3 and the reverse primer was H15240: 5′- TTATCTACYGAAAAGCCTCCTC−3′. These primers were designed to amplify DNA of the four species of elephantids, while excluding all other species whose sequences showed a total of 6 to 12 differential nucleotides in forward and reverse primer regions (Table 3). Four degenerate nucleotide bases were included in the primer sequences (3 in the L primer and 1 in the H primer) to allow amplification of DNA from both of the mtDNA clades of woolly mammoth^54–56^, both of the mtDNA clades of Asian elephant^57^, all the three mtDNA subclades of savanna elephants^30^ and all the five subclades of F-clade (which is found in all forest elephants but shows introgression into some savanna elephant populations)^30^. The degenerate positions were away from the 3′ end of the primer sequences and were not expected to adversely affect the overall specificity and/or sensitivity of the assay.

**Table 3:** Differential nucleotides within the primer region on Cyt *b* gene among 13 species used in this study.

Species	*n*	Forward primer L15123 (5′−3′)																							Reverse primer H15240 (5′−3′)																				
		T	A	C	T	A	C	T	R	A	T	Y	A	C	Y	A	T	A	G	C	C	A	C	T	T	A	T	C	T	A	C	Y	G	A	A	A	A	G	C	C	T	C	C	T	C
*E. maximus*	4	·	·	·	·	·	·	·	A	·	·	C/T	·	·	C	·	·	·	·	·	·	·	·	·	·	·	·	·	·	·	·	C	·	·	·	·	·	·	·	·	·	·	·	·	·
*L. africana*	8	·	·	·	·	·	·	·	A	·	·	C	·	·	C	·	·	·	·	·	·	·	·	·	·	·	·	·	·	·	·	C/T	·	·	·	·	·	·	·	·	·	·	·	·	·
*L. cyclotis*	9	·	·	·	·	·	·	·	A/G	·	·	C	·	·	C/T	·	·	·	·	·	·	·	·	·	·	·	·	·	·	·	·	C	·	·	·	·	·	·	·	·	·	·	·	·	·
*M. primigenius*	9	·	·	·	·	·	·	·	A	·	·	C	·	·	C	·	·	·	·	·	·	·	·	·	·	·	·	·	·	·	·	C/T	·	·	·	·	·	·	·	·	·	·	·	·	·
*H. amphibium*	2	·	·	·	·	·	·	·	C	·	C	A	·	·	C	·	·	·	·	·	T	·	·	·	·	G	·	·	·	·	·	G	·	·	·	·	·	·	·	·	·	·	·	·	·
*M. monoceros*	3	·	·	·	·	·	·	·	A	·	·	A	·	T	C	·	·	·	·	·	·	·	·	·	·	·	·	·	·	·	·	A	·	·	·	·	·	C	·	·	A	·	·	·	·
*P. aethiopicus*	3	·	·	·	·	·	·	·	C	·	C	T	G	T	T	·	·	·	·	·	A	·	·	·	·	G	·	·	·	·	·	A	·	·	G	·	·	A	·	·	C	·	·	·	·
*P. catodon*	3	·	C	·	·	·	A	·	C	·	C	A	G	T	A	·	·	·	·	·	·	·	·	·	·	·	·	·	·	·	·	G	·	·	G	·	·	A	·	·	G	·	·	·	·
*O. orca*	2	·	·	·	·	·	·	·	A	G	C	A	G	T	T	·	·	·	·	·	·	·	·	·	·	·	·	·	·	·	·	G	·	·	·	·	·	C	·	·	A	·	·	·	·
*O. rosmarus*	2	·	·	T	·	·	·	·	C	·	C	T	·	T	T	·	·	·	·	·	·	·	·	·	·	·	·	·	A	·	·	T	·	·	·	·	·	C	·	·	C	·	·	·	·
*C. simum*	3	·	·	·	·	·	T	·	C	·	C	T	C	T	A	·	·	·	·	·	·	·	·	·	·	·	·	·	A	·	·	G	·	·	·	·	·	T	·	·	·	·	·	·	·
*B. Taurus*	3	·	T	·	·	G	T	·	C	·	C	A	G	T	A	·	·	·	·	·	·	·	·	·	·	·	·	·	·	·	·	T	·	·	G	·	·	T	·	·	G	·	·	·	·
*H. sapiens*	1	·	C	·	·	G	·	·	T	G	C	A	·	·	T	·	·	·	·	·	A	·	·	C	·	G	·	·	·	·	·	T	·	·	G	T	·	·	·	·	·	·	·	·	·

*n* is the number of sequences from each species used for examining the differential nucleotides. Nucleotides identical to those in the primer sequences are shown as dots.

### Reproducibility tests

The primer pair L15123/H15240 was tested for reproducibility using PCRs of DNA extracts obtained from all of the elephantid species samples collected in the current study. PCRs for each amplification were carried out in a 50 µl reaction volume containing 5 µl DNA template of varied concentrations (10 ng/µl or less), 25 µl of 2 × EasyTaq^®^ PCR SuperMix (+dye) (Transgen, China), 0.2 µM of each primer, and deionized water to bring the final volume to 50 µl. Each set of PCRs was negatively controlled using deionized water. PCR cycling was performed on an Eppendorf Mastercycler ep thermocycler as follow: an initial denaturation at 95 °C for 3 minutes, 40 cycles of 30 s at 94 °C, 30 s at 51 °C, and 30 s at 72 °C; then a final extension for 20 mins at 72 °C. Amplified DNA products were purified using QIAquick PCR Purification Kit (Qiagen Inc.) and sequenced using Big Dye v3.1 on an ABI 3130xl Genetic Analyzer (Applied Biosystems, USA). Sequences were checked for quality and edited using SEQUENCHER version 5.2.4 (Gene Codes Corporation, USA). Primer sequences were trimmed from both termini of each sequence. The processed DNA sequences were then Blasted in GenBank using the NCBI nucleotide BLAST (blastn) program^58^ set at the default parameters to confirm the species correctness.

### Specificity tests

The primer pair L15123/H15240 was tested for its ability to exclude human and other non-target species. The primers L15123/H15240 were tested against the GenBank sequence database using the NCBI/Primer-BLAST program to identify non-target species the primer set might bind to *in silico*. Nineteen selected samples, including human (n = 5), domestic cattle (n = 4), common hippopotamus (n = 4) and white rhinoceros (n = 6) (samples listed in Supplementary Table S1), were used to perform empirical tests using the same PCR system and cycling program as above with DNA template concentrations of ~10 ng/µl. Moreover, mixed-sample experiments were performed to determine the specificity by including several DNA sources in an amplification system. DNA of mammoth ivory (MP27) and human hair follicle (H1), African savanna elephant dung (LA13) and cattle tissue (C4), and Asian elephant (EM11) dung and cattle tissue (C4) were mixed at concentration ratios of 1:1 and 1:5, respectively, and subjected to PCR amplification and sequencing as above.

### Resolution tests for elephantid species identification

Resolution tests were performed using sequences generated from the reproducibility tests section above. Six additional sequences of African forest elephant museum specimens^59^ downloaded from GenBank (Supplementary Table S3) and also included primer design in the section above, were made available for these tests. Nucleotide polymorphisms and nucleotide diversity (π) of the amplified Cyt *b* region were quantified using DnaSP v.3 software^60^. Pairwise comparisons were made to estimate interspecific divergence and intraspecific nucleotide diversity. Maximum Likelihood and Neighbour Joining inference of all the sequences was performed using MEGA 5.3, while Bayesian inference was done using MRBAYES version 3.2.6^61^. Two sequences (Accession: KY364233, EF632344) of American mastodon (*Mammut americanum*), a distance relative of the elephantids in the order Proboscidea, were used as an outgroup. The K2P nucleotide substitution model of evolution was selected using Bayesian Information Criterion (BIC) values computed using MEGA 5.3. Bayesian MCMC was run for 100 million generations: trees sampled every 1000 generations and convergence diagnostic calculated every 1000 generations. The convergence was achieved when convergence diagnostic value was ≤0.01. The phylogenetic tree was summarized in MrBayes after removing the first 25% of the tree as burn in. TRACER version 1.6^62^, was used to examine the convergence diagnostics from Bayesian phylogenetic analyses, and results were considered when the effective sample size (ESS) was above a threshold of 200. Maximum likelihood analysis was conducted using the nearest neighbour interchange heuristic method using 1000 bootstrap replicates. Maximum composite likelihood model containing uniform distributed rates among the sites with 1000 replicates was used in Neighbour Joining analysis. The phylogenetic trees were finally visualized using FIGTREE version 1.4.3^63^.

Pairwise genetic distances of individuals within species (intra-*d*) and among species (inter-*d*) were compared using Kimura’s (1980) two parameter (K2P) model^64^ and the *p*-distance model implemented in MEGA 5.3. Our previous study showed transition substitution (Ts) was the optimal substitution type to visualize the gap between intra-*d* and inter-*d* for the Cyt *b* gene fragment^65^. In this study, K2P and *p*-distance were all computed using Ts substitution and uniform distributed rates among sites, with 1000 bootstrap replicates. Matrices comprising a range of sequence genetic distances between the four elephantid species (inter-*d*), and within each species (intra-*d*), was constructed to demonstrate the sequence divergence at this Cyt *b* region. The means and standard deviations (*SD*) of intra-*d* and inter-*d* were calculated using the program SPSS 19.0.0 (IBM Corp, NY, US).

### Sensitivity tests

DNA recovered from an ivory sample of woolly mammoth and two faecal samples of an Asian and an African savanna elephant were serially diluted from ~10 ng/µl to ~1 ng/µl, ~0.1 ng/µl, ~10 pg/µl and 1 pg/µl, and used to test the sensitivity of our PCR system. For African forest elephant DNA (ivory DNA), only the four latter serial dilutions of DNA were included in this test because the total quantity of isolated DNA was <10 ng/µl. 25 µl reaction volume and 2 µl total DNA of each dilution level were used. The amplicons were separated on a 1.5% agarose gel and visualized under UV.

Absolute quantification using qPCR was performed to determine the actual copy numbers of the target mtDNA in the samples outlined above^66^. The target PCR products of an Asian elephant were excised from gel and purified using AxyPrep DNA gel extraction kit (Axygen, USA). Purified products were inserted into cloning vector pMD18-T (Takara, Japan), transformed into competent *Escherichia coli* XL-10 Gold cells, plated on LB agar plates containing ampicillin (100 μg/ml) and X-gal/IPTG, and grown at 37 °C overnight. Single white colonies were randomly picked and plasmids extracted using Plasmid Mini-prep Kit (TsingKe BioTech, China). The presence of the desired fragment size in the clones was confirmed *via* PCR and sequencing on an ABI 3130xl Genetic Analyzer (Applied Biosystems, USA). One candidate clone was quantified using Nanodrop spectrophotometer (ND-1000, NanoDrop Technologies) and used as the standard. The mtDNA copy numbers for the standard were calculated as previously described^67^. For the qPCR, the standard DNA (template) was first serially diluted in deionized water (from 10^8^ to 10^1^ specific copies/5 μl). Five serial dilutions from 10^6^ to 10^2^ (copies/5 μl) were used to create a standard curve. The copy number of effective template was tested for initial input DNA (~10 ng/µl, except for forest elephant that started with 1 ng/µl) and the least quantity of DNA that produced visible bands (~1 pg/µl) from all the four elephantid species. qPCRs were carried out in a 20 μl reaction volume containing 1 μl of DNA dilution samples, 0.8 µM of each primer, 10ul TB Green^TM^ Premix Ex Taq^TM^ II (TaKaRa, Japan) and deionized water. All the reactions were run in triplicate on a CFX 384 thermocycler (Bio-rad Laboratories, Hercules, CA, USA). The cycling conditions were as follows: 2 min at 95 °C, followed by 40 cycles of 95 °C for 15 s and 51 °C for 30 s. The Bio-Rad CFX Manager software (version 1.6) was used for calculation of the starting quantity of the amplified DNA.

### Ethics statement

All the fecal samples were non-invasively collected from zoo animals. Elephant ivory and domestic cattle tissue samples were provided by and the State Forestry and Grassland Administration Detecting Center of Wildlife of China, and woolly mammoth ivory samples were provided by carving factories in China. The human hair samples were obtained with the informed consent of all the participants. All the experiments were carried out in accordance with the relevant guidelines and regulations of Northeast Forestry University, and the protocols were approved by Ethical Review Board of Northeast Forestry University (Approval Number: 2017003).

## Supplementary information

Supplementary FigureS1, TableS1, TableS2, TableS3

## Data Availability

A total of 150 partial cytochrome *b* sequences generated during the current study were deposited in the GenBank https://www.ncbi.nlm.nih.gov/ with Accession Numbers MN230722-MN230871. These sequences are also available in CNGB (https://db.cngb.org/) with Accession Numbers N_000000086-N_000000235.
