# Supplementary material for: Distinguishing extant elephants ivory from mammoth ivory using a short sequence of cytochrome b gene
Source: Sci Rep. 2019 Dec 11;9:18863. doi: 10.1038/s41598-019-55094-x (PMC6906310; doi:10.1038/s41598-019-55094-x)

**Distinguishing extant elephants ivory from mammoth ivory using a short sequence of cytochrome b gene**

Jacob Njaramba Ngatia^1^, Tian Ming Lan^2, 3, 4^, Yue Ma^1, 5^, Thi Dao Dinh^1^, Zhen Wang^1, 5^, Thomas D. Dahmer^6^, Yan Chun Xu^1, 5,7^*

^1^ College of Wildlife and Protected Areas, Northeast Forestry University, Harbin, 150040, China

^2^ BGI - Shenzhen, Shenzhen, 518083, China

^3^ Laboratory of Genomics and Molecular Biomedicine, Department of Biology, University of Copenhagen, Copenhagen DK-2100, Denmark.

^4^ China National GeneBank, BGI - Shenzhen, Shenzhen, 518083, China.

^5^ State Forestry and Grassland Administration Detecting Center of Wildlife, Harbin, 150040, China

^6^ Ecosystems Ltd, No. 40 Shek Pai Wan Road, Aberdeen, Hong Kong, China

^7^State Forestry and Grassland Administration Research Center of Engineering Technology for Wildlife Conservation and Utilization of China, Harbin, 150040, China

**Correspondence**: [xu_daniel@163.com](mailto:xu_daniel@163.com)

Figure caption.

**Figure S1.** Agarose gel electropherogram of the sensitivity test. DNA extracted from an Asian elephant (#EM35), African savanna elephant (#LA10), and woolly mammoth (#MP5) was diluted in ~10 ng/µl (D1), ~1 ng/µl (D2), ~0.1 ng/µl (D3), ~10pg/µl (D4) and 1 pg/µl (D5), while for African forest elephant (#LC4), four latter dilutions of DNA were done followed by amplification in triplicate through PCR using the L15123/ H15240 primers.

| **Sample Name** | **Species** | **Nomenclature** | **Sample Type** | **Sample Source** | **Sampling site** | **Geographic origin of the animals involved** | **PCR success and species correctly identified** |
| --- | --- | --- | --- | --- | --- | --- | --- |
| L1 | African Savanna elephant | *Loxodonta africana* | Feces | Zoo collection | Zhengzhou Zoo | South Africa | YES |
| L2 |  | *Loxodonta africana* | Feces | Zoo collection | Zhengzhou Zoo | South Africa | YES |
| L3 |  | *Loxodonta africana* | Feces | Zoo collection | Hangzhou Safari Park | Zimbabwe | YES |
| L4 |  | *Loxodonta africana* | Feces | Zoo collection | Hangzhou Safari Park | Zimbabwe | YES |
| L5 |  | *Loxodonta africana* | Feces | Zoo collection | Hangzhou Safari Park | Zimbabwe | YES |
| L6 |  | *Loxodonta africana* | Feces | Zoo collection | Guangzhou Zoo | South Africa | YES |
| L7 |  | *Loxodonta africana* | Feces | Zoo collection | Luoyang Zoo | South Africa | YES |
| L8 |  | *Loxodonta africana* | Feces | Zoo collection | Luoyang Zoo | South Africa | YES |
| L9 |  | *Loxodonta africana* | Feces | Zoo collection | Beijing Zoo | Not established | YES |
| L10 |  | *Loxodonta africana* | Feces | Zoo collection | Beijing Zoo | Not established | YES |
| L11 |  | *Loxodonta africana* | Feces | Zoo collection | Beijing Zoo | Not established | YES |
| L12 |  | *Loxodonta africana* | Feces | Zoo collection | Shanghai Wild Animal Park | Zimbabwe | YES |
| L13 |  | *Loxodonta africana* | Feces | Zoo collection | Shanghai Wild Animal Park | Zimbabwe | YES |
| L14 |  | *Loxodonta africana* | Feces | Zoo collection | Shanghai Wild Animal Park | Zimbabwe | YES |
| L15 |  | *Loxodonta africana* | Feces | Zoo collection | Shanghai Wild Animal Park | Zimbabwe | YES |
| L16 |  | *Loxodonta africana* | Feces | Zoo collection | Shanghai Wild Animal Park | Zimbabwe | YES |
| L17 |  | *Loxodonta africana* | Feces | Zoo collection | Shanghai Wild Animal Park | Zimbabwe | YES |
| L18 |  | *Loxodonta africana* | Feces | Zoo collection | Shanghai Wild Animal Park | Zimbabwe | YES |
| L19 |  | *Loxodonta africana* | Feces | Zoo collection | Shanghai Wild Animal Park | Zimbabwe | YES |
| L20 |  | *Loxodonta africana* | Feces | Zoo collection | Shanghai Wild Animal Park | Zimbabwe | YES |
| L21 |  | *Loxodonta africana* | Ivory | Laboratory collection | Northeast Forestry University | Not established | YES |
| L22 |  | *Loxodonta africana* | Ivory | Laboratory collection | Northeast Forestry University | Not established | YES |

Table S1. Sample details of specimens used in this study and their species identification success. Geographical origin of some animals could not be established.

| **Sample Name** | **Species** | **Nomenclature** | **Sample Type** | **Sample Source** | **Sampling site** | **Geographic origin of the animals involved** | **PCR success and species correctly identified** |
| --- | --- | --- | --- | --- | --- | --- | --- |
| LC1 | African Forest Elephant | *Loxodonta cyclotis* | Ivory | Laboratory collection | Northeast Forestry University | Not established | YES |
| LC2 |  | *Loxodonta cyclotis* | Ivory | Laboratory collection | Northeast Forestry University | Not established | YES |
| LC3 |  | *Loxodonta cyclotis* | Ivory | Laboratory collection | Northeast Forestry University | Not established | YES |
| LC4 |  | *Loxodonta cyclotis* | Ivory | Laboratory collection | Northeast Forestry University | Not established | YES |
| LC5 |  | *Loxodonta cyclotis* | Ivory | Laboratory collection | Northeast Forestry University | Not established | YES |
| LC6 |  | *Loxodonta cyclotis* | Ivory | Laboratory collection | Northeast Forestry University | Not established | YES |
| LC7 |  | *Loxodonta cyclotis* | Ivory | Laboratory collection | Northeast Forestry University | Not established | YES |
| LC8 |  | *Loxodonta cyclotis* | Ivory | Laboratory collection | Northeast Forestry University | Not established | YES |
|  |  |  |  |  |  |  |  |
| EM1 | Asian Elephant | *Elephas maximus* | Feces | Zoo collection | Hangzhou Safari Park | Thailand | YES |
| EM2 |  | *Elephas maximus* | Feces | Zoo collection | Hangzhou Safari Park | Thailand | YES |
| EM3 |  | *Elephas maximus* | Feces | Zoo collection | Hangzhou Safari Park | Thailand | YES |
| EM4 |  | *Elephas maximus* | Feces | Zoo collection | Guangzhou Zoo | Hainan, China | YES |
| EM5 |  | *Elephas maximus* | Feces | Zoo collection | Guangzhou Zoo | Not established | YES |
| EM6 |  | *Elephas maximus* | Feces | Zoo collection | Guangzhou Zoo | Not established | YES |
| EM7 |  | *Elephas maximus* | Feces | Zoo collection | Nanjing Zoo | Yunnan, China | YES |
| EM8 |  | *Elephas maximus* | Feces | Zoo collection | Hefei Wildlife Zoo | Not established | YES |
| EM9 |  | *Elephas maximus* | Feces | Zoo collection | Shanghai Wild Animal Park | Laos | YES |
| EM10 |  | *Elephas maximus* | Feces | Zoo collection | Shanghai Wild Animal Park | Yunnan, China | YES |
| EM11 |  | *Elephas maximus* | Feces | Zoo collection | Shanghai Wild Animal Park | Laos | YES |
| EM12 |  | *Elephas maximus* | Feces | Zoo collection | Shanghai Wild Animal Park | Laos | YES |
| EM13 |  | *Elephas maximus* | Feces | Zoo collection | Shanghai Wild Animal Park | Laos | YES |
| EM14 |  | *Elephas maximus* | Feces | Zoo collection | Shanghai Wild Animal Park | Laos | YES |
| EM15 |  | *Elephas maximus* | Feces | Zoo collection | Shanghai Wild Animal Park | Laos | YES |
| EM16 |  | *Elephas maximus* | Feces | Zoo collection | Shanghai Wild Animal Park | Laos | YES |
| EM17 |  | *Elephas maximus* | Feces | Zoo collection | Shanghai Wild Animal Park | Laos | YES |
| EM18 |  | *Elephas maximus* | Feces | Zoo collection | Shanghai Wild Animal Park | Laos | YES |

| **Sample Name** | **Species** | **Nomenclature** | **Sample Type** | **Sample Source** | **Sampling site** | **Geographic origin of the animals involved** | **PCR success and species correctly identified** |
| --- | --- | --- | --- | --- | --- | --- | --- |
| EM19 | Asian Elephant | *Elephas maximus* | Feces | Zoo collection | Kunming Zoo | Yunan, China | YES |
| EM20 |  | *Elephas maximus* | Feces | Zoo collection | Kunming Zoo | Yunan, China | YES |
| EM21 |  | *Elephas maximus* | Feces | Zoo collection | Shanghai Wild Animal Park | China | YES |
| EM22 |  | *Elephas maximus* | Feces | Zoo collection | Shanghai Wild Animal Park | Not established | YES |
| EM23 |  | *Elephas maximus* | Feces | Zoo collection | Lanzhou Zoo | China | YES |
| EM24 |  | *Elephas maximus* | Feces | Zoo collection | Qinhuangdao Safari Park | Myanmar | YES |
| EM25 |  | *Elephas maximus* | Feces | Zoo collection | Qinhuangdao Safari Park | Myanmar | YES |
| EM26 |  | *Elephas maximus* | Feces | Zoo collection | Qinhuangdao Safari Park | Myanmar | YES |
| EM27 |  | *Elephas maximus* | Feces | Zoo collection | Dalian Forestry Zoo | Thailand | YES |
| EM28 |  | *Elephas maximus* | Feces | Zoo collection | Dalian Forestry Zoo | Thailand | YES |
| EM29 |  | *Elephas maximus* | Feces | Zoo collection | Dalian Forestry Zoo | Thailand | YES |
| EM30 |  | *Elephas maximus* | Feces | Zoo collection | Dalian Forestry Zoo | Thailand | YES |
| EM31 |  | *Elephas maximus* | Feces | Zoo collection | Dalian Forestry Zoo | Hunan , China | YES |
| EM32 |  | *Elephas maximus* | Feces | Zoo collection | Dalian Forestry Zoo | Thailand | YES |
| EM33 |  | *Elephas maximus* | Feces | Zoo collection | Dalian Forestry Zoo | Thailand | YES |
| EM34 |  | *Elephas maximus* | Feces | Zoo collection | Safari Park Shenzhen | Jilin, China | YES |
| EM35 |  | *Elephas maximus* | Feces | Zoo collection | Safari Park Shenzhen | Myanmar | YES |
| EM36 |  | *Elephas maximus* | Feces | Zoo collection | Safari Park Shenzhen | Myanmar | YES |
| EM37 |  | *Elephas maximus* | Feces | Zoo collection | Safari Park Shenzhen | Myanmar | YES |
| EM38 |  | *Elephas maximus* | Feces | Zoo collection | Safari Park Shenzhen | Myanmar | YES |
| EM39 |  | *Elephas maximus* | Feces | Zoo collection | Safari Park Shenzhen | Not established | YES |
| EM40 |  | *Elephas maximus* | Feces | Zoo collection | Safari Park Shenzhen | Not established | YES |
| EM41 |  | *Elephas maximus* | Feces | Zoo collection | Safari Park Shenzhen | Not established | YES |
| EM42 |  | *Elephas maximus* | Feces | Zoo collection | Safari Park Shenzhen | Not established | YES |
| EM43 |  | *Elephas maximus* | Feces | Zoo collection | Safari Park Shenzhen | Not established | YES |
| EM44 |  | *Elephas maximus* | Feces | Zoo collection | Safari Park Shenzhen | Myanmar | YES |
| EM45 |  | *Elephas maximus* | Feces | Zoo collection | Wenzhou Zoo | Not established | YES |
| **Sample Name** | **Species** | **Nomenclature** | **Sample Type** | **Sample Source** | **Sampling site** | **Geographic origin of the animals involved** | **PCR success and species correctly identified** |
| EM46 | Asian Elephant | *Elephas maximus* | Feces | Zoo collection | Xi’an Wildlife Zoo | Yunan, China | YES |
| EM47 |  | *Elephas maximus* | Feces | Zoo collection | Fuzhou Zoo | Not established | YES |
| EM48 |  | *Elephas maximus* | Feces | Zoo collection | Fuzhou Zoo | Not established | YES |
| EM49 |  | *Elephas maximus* | Feces | Zoo collection | Dalian Forest Zoo | Thailand | YES |
| EM50 |  | *Elephas maximus* | Feces | Zoo collection | Beijing Zoo | Not established | YES |
| EM51 |  | *Elephas maximus* | Feces | Zoo collection | Beijing Zoo | Not established | YES |
| EM52 |  | *Elephas maximus* | Feces | Zoo collection | Beijing Zoo | Not established | YES |
| EM53 |  | *Elephas maximus* | Feces | Zoo collection | Beijing Zoo | Not established | YES |
| EM54 |  | *Elephas maximus* | Feces | Zoo collection | Beijing Zoo | Not established | YES |
| EM55 |  | *Elephas maximus* | Feces | Zoo collection | Shanghai Wild Animal Park | Laos | YES |
| EM56 |  | *Elephas maximus* | Feces | Zoo collection | Chengdu Zoo | Yunnan, China | YES |
| EM57 |  | *Elephas maximus* | Feces | Zoo collection | Chongqing Zoo | Not established | YES |
| EM58 |  | *Elephas maximus* | Feces | Zoo collection | Chongqing Zoo | Not established | YES |
| EM59 |  | *Elephas maximus* | Feces | Zoo collection | Chongqing Zoo | Not established | YES |
| EM60 |  | *Elephas maximus* | Feces | Zoo collection | Shanghai Wild Animal Park | Myanmar | YES |
| EM61 |  | *Elephas maximus* | Feces | Zoo collection | Shanghai Wild Animal Park | Myanmar | YES |
|  |  |  |  |  |  |  |  |
| MP1 | Woolly Mammoth | *Mammuthus primigenius* | Ivory | Carving factory collection | Guangzhou, China | Yakutia, Russia | YES |
| MP2 |  | *Mammuthus primigenius* | Ivory | Carving factory collection | Guangzhou, China | Yakutia, Russia | YES |
| MP3 |  | *Mammuthus primigenius* | Ivory | Carving factory collection | Guangzhou, China | Yakutia, Russia | YES |
| MP4 |  | *Mammuthus primigenius* | Ivory | Carving factory collection | Guangzhou, China | Yakutia, Russia | YES |
| MP5 |  | *Mammuthus primigenius* | Ivory | Carving factory collection | Guangzhou, China | Yakutia, Russia | YES |
| MP6 |  | *Mammuthus primigenius* | Ivory | Carving factory collection | Guangzhou, China | Yakutia, Russia | YES |
| MP7 |  | *Mammuthus primigenius* | Ivory | Carving factory collection | Guangzhou, China | Yakutia, Russia | YES |
| MP8 |  | *Mammuthus primigenius* | Ivory | Carving factory collection | Guangzhou, China | Yakutia, Russia | YES |
| MP9 |  | *Mammuthus primigenius* | Ivory | Carving factory collection | Guangzhou, China | Yakutia, Russia | YES |
| MP10 |  | *Mammuthus primigenius* | Ivory | Carving factory collection | Guangzhou, China | Yakutia, Russia | YES |

| **Sample Name** | **Species** | | **Nomenclature** | **Sample Type** | **Sample Source** | **Sampling site** | **Geographic origin of the animals involved** | **PCR success and species correctly identified** |
| --- | --- | --- | --- | --- | --- | --- | --- | --- |
| MP11 | Woolly Mammoth | | *Mammuthus primigenius* | Ivory | Carving factory collection | Guangzhou, China | Yakutia, Russia | YES |
| MP12 |  | | *Mammuthus primigenius* | Ivory | Carving factory collection | Guangzhou, China | Yakutia, Russia | YES |
| MP13 |  | | *Mammuthus primigenius* | Ivory | Carving factory collection | Guangzhou, China | Yakutia, Russia | YES |
| MP14 |  | | *Mammuthus primigenius* | Ivory | Carving factory collection | Guangzhou, China | Yakutia, Russia | YES |
| MP15 |  | | *Mammuthus primigenius* | Ivory | Carving factory collection | Guangzhou, China | Yakutia, Russia | YES |
| MP16 |  | | *Mammuthus primigenius* | Ivory | Carving factory collection | Guangzhou, China | Yakutia, Russia | YES |
| MP17 |  | | *Mammuthus primigenius* | Ivory | Carving factory collection | Guangzhou, China | Yakutia, Russia | YES |
| MP18 |  | | *Mammuthus primigenius* | Ivory | Carving factory collection | Guangzhou, China | Yakutia, Russia | YES |
| MP19 |  | | *Mammuthus primigenius* | Ivory | Carving factory collection | Guangzhou, China | Yakutia, Russia | YES |
| MP20 |  | | *Mammuthus primigenius* | Ivory | Carving factory collection | Guangzhou, China | Yakutia, Russia | YES |
| MP21 |  | | *Mammuthus primigenius* | Ivory | Carving factory collection | Guangzhou, China | Yakutia, Russia | YES |
| MP22 |  | | *Mammuthus primigenius* | Ivory | Carving factory collection | Guangzhou, China | Yakutia, Russia | YES |
| MP23 |  | | *Mammuthus primigenius* | Ivory | Carving factory collection | Guangzhou, China | Yakutia, Russia | YES |
| MP24 |  | | *Mammuthus primigenius* | Ivory | Carving factory collection | Guangzhou, China | Yakutia, Russia | YES |
| MP25 |  | | *Mammuthus primigenius* | Ivory | Carving factory collection | Guangzhou, China | Yakutia, Russia | YES |
| MP26 |  | | *Mammuthus primigenius* | Ivory | Carving factory collection | Guangzhou, China | Yakutia, Russia | YES |
| MP27 |  | | *Mammuthus primigenius* | Ivory | Carving factory collection | Guangzhou, China | Yakutia, Russia | YES |
| MP28 |  | | *Mammuthus primigenius* | Ivory | Carving factory collection | Guangzhou, China | Yakutia, Russia | YES |
| MP29 |  | | *Mammuthus primigenius* | Ivory | Carving factory collection | Guangzhou, China | Yakutia, Russia | YES |
| MP30 |  | | *Mammuthus primigenius* | Ivory | Carving factory collection | Guangzhou, China | Yakutia, Russia | YES |
| MP31 |  | | *Mammuthus primigenius* | Ivory | Carving factory collection | Guangzhou, China | Yakutia, Russia | YES |
| MP32 |  | | *Mammuthus primigenius* | Ivory | Carving factory collection | Guangzhou, China | Yakutia, Russia | YES |
| MP33 |  | | *Mammuthus primigenius* | Ivory | Carving factory collection | Guangzhou, China | Yakutia, Russia | YES |
| MP34 |  | | *Mammuthus primigenius* | Ivory | Carving factory collection | Guangzhou, China | Yakutia, Russia | YES |
| MP35 |  | | *Mammuthus primigenius* | Ivory | Carving factory collection | Guangzhou, China | Yakutia, Russia | YES |
| MP36 |  | | *Mammuthus primigenius* | Ivory | Carving factory collection | Guangzhou, China | Yakutia, Russia | YES |
| **Sample Name** | **Species** | **Nomenclature** | | **Sample Type** | **Sample Source** | **Sampling site** | **Geographic origin of the animals involved** | **PCR success and species correctly identified** |
| MP37 | Woolly Mammoth | *Mammuthus primigenius* | | Ivory | Carving factory collection | Guangzhou, China | Yakutia, Russia | YES |
| MP38 |  | *Mammuthus primigenius* | | Ivory | Carving factory collection | Guangzhou, China | Yakutia, Russia | YES |
| MP39 |  | *Mammuthus primigenius* | | Ivory | Carving factory collection | Guangzhou, China | Yakutia, Russia | YES |
| MP40 |  | *Mammuthus primigenius* | | Ivory | Carving factory collection | Guangzhou, China | Yakutia, Russia | YES |
| MP41 |  | *Mammuthus primigenius* | | Ivory | Carving factory collection | Guangzhou, China | Yakutia, Russia | YES |
| MP42 |  | *Mammuthus primigenius* | | Ivory | Carving factory collection | Guangzhou, China | Yakutia, Russia | YES |
| MP43 |  | *Mammuthus primigenius* | | Ivory | Carving factory collection | Guangzhou, China | Yakutia, Russia | YES |
| MP44 |  | *Mammuthus primigenius* | | Ivory | Carving factory collection | Guangzhou, China | Yakutia, Russia | YES |
| MP45 |  | *Mammuthus primigenius* | | Ivory | Carving factory collection | Guangzhou, China | Yakutia, Russia | YES |
| MP46 |  | *Mammuthus primigenius* | | Ivory | Carving factory collection | Guangzhou, China | Yakutia, Russia | YES |
| MP47 |  | *Mammuthus primigenius* | | Ivory | Carving factory collection | Guangzhou, China | Yakutia, Russia | YES |
| MP48 |  | *Mammuthus primigenius* | | Ivory | Carving factory collection | Guangzhou, China | Yakutia, Russia | YES |
| MP49 |  | *Mammuthus primigenius* | | Ivory | Carving factory collection | Guangzhou, China | Yakutia, Russia | YES |
| MP50 |  | *Mammuthus primigenius* | | Ivory | Carving factory collection | Guangzhou, China | Yakutia, Russia | YES |
| MP51 |  | *Mammuthus primigenius* | | Ivory | Carving factory collection | Guangzhou, China | Yakutia, Russia | YES |
| MP52 |  | *Mammuthus primigenius* | | Ivory | Carving factory collection | Guangzhou, China | Yakutia, Russia | YES |
| MP53 |  | *Mammuthus primigenius* | | Ivory | Carving factory collection | Guangzhou, China | Yakutia, Russia | YES |
| MP54 |  | *Mammuthus primigenius* | | Ivory | Carving factory collection | Guangzhou, China | Yakutia, Russia | YES |
| MP55 |  | *Mammuthus primigenius* | | Ivory | Carving factory collection | Guangzhou, China | Yakutia, Russia | YES |
| MP56 |  | *Mammuthus primigenius* | | Ivory | Carving factory collection | Guangzhou, China | Yakutia, Russia | YES |
| MP57 |  | *Mammuthus primigenius* | | Ivory | Carving factory collection | Guangzhou, China | Yakutia, Russia | YES |
| MP58 |  | *Mammuthus primigenius* | | Ivory | Carving factory collection | Guangzhou, China | Yakutia, Russia | YES |
| MP59 |  | *Mammuthus primigenius* | | Ivory | Carving factory collection | Guangzhou, China | Yakutia, Russia | YES |
| MP60 |  | *Mammuthus primigenius* | | Ivory | Carving factory collection | Guangzhou, China | Yakutia, Russia | NO |
| MP61 |  | *Mammuthus primigenius* | | Ivory | Carving factory collection | Guangzhou, China | Yakutia, Russia | NO |

| **Sample Name** | **Species** | **Nomenclature** | **Sample Type** | **Sample Source** | **Sampling site** | **Geographic origin of the animals involved** | **PCR success and species correctly identified** |
| --- | --- | --- | --- | --- | --- | --- | --- |
|  |  |  |  |  |  |  |  |
| H1 | Human | *Homo sapiens* | Hair | Volunteer | Northeast forestry university | China | NO |
| H2 |  | *Homo sapiens* | Hair | Volunteer | Northeast forestry university | China | NO |
| H3 |  | *Homo sapiens* | Hair | Volunteer | Northeast forestry university | China | NO |
| H4 |  | *Homo sapiens* | Hair | Volunteer | Northeast forestry university | China | NO |
| H5 |  | *Homo sapiens* | Hair | Volunteer | Northeast forestry university | China | NO |
|  |  |  |  |  |  |  |  |
| HP1 | Hippopotamus | *Hippopotamus amphibius* | Feces | Zoo collection | Shanghai Wild Animal Park | Not established | NO |
| HP2 |  | *Hippopotamus amphibius* | Feces | Zoo collection | Shanghai Wild Animal Park | Not established | NO |
| HP3 |  | *Hippopotamus amphibius* | Feces | Zoo collection | Shanghai Wild Animal Park | Not established | NO |
| HP4 |  | *Hippopotamus amphibius* | Feces | Zoo collection | Shanghai Wild Animal Park | Not established | NO |
|  |  |  |  |  |  |  |  |
| WR1 | White rhinoceros | *Ceratotherium simum* | Feces | Zoo collection | Shanghai Wild Animal Park | South Africa | NO |
| WR2 |  | *Ceratotherium simum* | Feces | Zoo collection | Shanghai Wild Animal Park | South Africa | NO |
| WR3 |  | *Ceratotherium simum* | Feces | Zoo collection | Shanghai Wild Animal Park | South Africa | NO |
| WR4 |  | *Ceratotherium simum* | Feces | Zoo collection | Shanghai Wild Animal Park | South Africa | NO |
| WR5 |  | *Ceratotherium simum* | Feces | Zoo collection | Shanghai Wild Animal Park | South Africa | NO |
| WR6 |  | *Ceratotherium simum* | Feces | Zoo collection | Shanghai Wild Animal Park | South Africa | NO |
|  |  |  |  |  |  |  |  |
| C1 | Domestic cow | *Bos taurus* | tissue | Laboratory collection | Northeast forestry university | China | NO |
| C2 |  | *Bos taurus* | tissue | Laboratory collection | Northeast forestry university | China | NO |
| C3 |  | *Bos taurus* | tissue | Laboratory collection | Northeast forestry university | China | NO |
| C4 |  | *Bos taurus* | tissue | Laboratory collection | Northeast forestry university | China | NO |

**Table S2**. Similarity matches of DNA sequences from this study with the reference sequences in NCBI GenBank. E- value indicates the expected value, while % NS indicates the percent nucleotide similarity.

| **Sample Name** | **Species** | **E value** | **% NS** | **Matched species (Accession No)** |
| --- | --- | --- | --- | --- |
| LA1 | *Loxodonta africana* | 9.00E-52 | 100.00% | [JQ438758.1](https://www.ncbi.nlm.nih.gov/nucleotide/JQ438758.1?report=genbank&log$=nucltop&blast_rank=1&RID=91FDG2GU014) |
| LA2 |  | 9.00E-52 | 100.00% | [JQ438758.1](https://www.ncbi.nlm.nih.gov/nucleotide/JQ438758.1?report=genbank&log$=nucltop&blast_rank=1&RID=DX51CEKJ014) |
| LA3 |  | 9.00E-52 | 100.00% | [JQ438674.1](https://www.ncbi.nlm.nih.gov/nucleotide/JQ438674.1?report=genbank&log$=nucltop&blast_rank=1&RID=DX4YJVTT014) |
| LA4 |  | 9.00E-52 | 100.00% | [JQ438746.1](https://www.ncbi.nlm.nih.gov/nucleotide/JQ438746.1?report=genbank&log$=nucltop&blast_rank=2&RID=DX53PU2C014) |
| LA5 |  | 9.00E-52 | 100.00% | [JQ438674.1](https://www.ncbi.nlm.nih.gov/nucleotide/JQ438674.1?report=genbank&log$=nucltop&blast_rank=1&RID=DX568208014) |
| LA6 |  | 9.00E-52 | 100.00% | [JQ438674.1](https://www.ncbi.nlm.nih.gov/nucleotide/JQ438674.1?report=genbank&log$=nucltop&blast_rank=1&RID=DX5B023H015) |
| LA7 |  | 9.00E-52 | 100.00% | [JQ438674.1](https://www.ncbi.nlm.nih.gov/nucleotide/JQ438674.1?report=genbank&log$=nucltop&blast_rank=1&RID=DX5DHXVV015) |
| LA8 |  | 9.00E-52 | 100.00% | [JQ438674.1](https://www.ncbi.nlm.nih.gov/nucleotide/JQ438674.1?report=genbank&log$=nucltop&blast_rank=1&RID=DX5FZZCT014) |
| LA9 |  | 9.00E-52 | 100.00% | [JQ438758.1](https://www.ncbi.nlm.nih.gov/nucleotide/JQ438758.1?report=genbank&log$=nucltop&blast_rank=1&RID=DX5KE3MY014) |
| LA10 |  | 9.00E-52 | 100.00% | [JQ438758.1](https://www.ncbi.nlm.nih.gov/nucleotide/JQ438758.1?report=genbank&log$=nucltop&blast_rank=1&RID=DX5NRU3M014) |
| LA11 |  | 9.00E-52 | 100.00% | [JQ438674.1](https://www.ncbi.nlm.nih.gov/nucleotide/JQ438674.1?report=genbank&log$=nucltop&blast_rank=1&RID=DX5S31AB015) |
| LA12 |  | 9.00E-52 | 100.00% | [JQ438674.1](https://www.ncbi.nlm.nih.gov/nucleotide/JQ438674.1?report=genbank&log$=nucltop&blast_rank=1&RID=DX5UMA9E015) |
| LA13 |  | 9.00E-52 | 100.00% | [JQ438674.1](https://www.ncbi.nlm.nih.gov/nucleotide/JQ438674.1?report=genbank&log$=nucltop&blast_rank=1&RID=DX639KXW014) |
| LA14 |  | 9.00E-52 | 100.00% | [JQ438674.1](https://www.ncbi.nlm.nih.gov/nucleotide/JQ438674.1?report=genbank&log$=nucltop&blast_rank=1&RID=DX69FD5W014) |
| LA15 |  | 9.00E-52 | 100.00% | [JQ438674.1](https://www.ncbi.nlm.nih.gov/nucleotide/JQ438674.1?report=genbank&log$=nucltop&blast_rank=1&RID=DX6EVUDT015) |
| LA16 |  | 9.00E-52 | 100.00% | [JQ438674.1](https://www.ncbi.nlm.nih.gov/nucleotide/JQ438674.1?report=genbank&log$=nucltop&blast_rank=1&RID=DX6JCCJH015) |
| LA17 |  | 9.00E-52 | 100.00% | [JQ438674.1](https://www.ncbi.nlm.nih.gov/nucleotide/JQ438674.1?report=genbank&log$=nucltop&blast_rank=1&RID=DX6P7K6F015) |
| LA18 |  | 9.00E-52 | 100.00% | [JQ438674.1](https://www.ncbi.nlm.nih.gov/nucleotide/JQ438674.1?report=genbank&log$=nucltop&blast_rank=1&RID=DX6VDA7G014) |
| LA19 |  | 9.00E-52 | 100.00% | [JQ438758.1](https://www.ncbi.nlm.nih.gov/nucleotide/JQ438758.1?report=genbank&log$=nucltop&blast_rank=1&RID=DX6Z43NT015) |
| LA20 |  | 9.00E-52 | 100.00% | [JQ438758.1](https://www.ncbi.nlm.nih.gov/nucleotide/JQ438758.1?report=genbank&log$=nucltop&blast_rank=1&RID=DX748G83014) |
| LA21 |  | 9.00E-52 | 100.00% | [JQ438674.1](https://www.ncbi.nlm.nih.gov/nucleotide/JQ438674.1?report=genbank&log$=nucltop&blast_rank=1&RID=DX7BPND5014) |
| LA22 |  | 9.00E-52 | 100.00% | [JQ438758.1](https://www.ncbi.nlm.nih.gov/nucleotide/JQ438758.1?report=genbank&log$=nucltop&blast_rank=1&RID=DX7FBF36014) |
|  |  |  |  |  |
| LC1 | *Loxodonta cyclotis* | 9.00E-52 | 100.00% | [KY616975.1](https://www.ncbi.nlm.nih.gov/nucleotide/KY616975.1?report=genbank&log$=nucltop&blast_rank=1&RID=DX7KYM60014) |
| LC2 |  | 9.00E-52 | 100.00% | [KY616981.1](https://www.ncbi.nlm.nih.gov/nucleotide/KY616981.1?report=genbank&log$=nucltop&blast_rank=1&RID=DX8BESG6014) |
| LC3 |  | 9.00E-52 | 100.00% | [KY616981.1](https://www.ncbi.nlm.nih.gov/nucleotide/KY616981.1?report=genbank&log$=nucltop&blast_rank=1&RID=DX8FMWJ7014) |
| LC4 |  | 9.00E-52 | 100.00% | [KY616981.1](https://www.ncbi.nlm.nih.gov/nucleotide/KY616981.1?report=genbank&log$=nucltop&blast_rank=1&RID=DX8MCUSP01R) |
| LC5 |  | 9.00E-52 | 100.00% | [KY616981.1](https://www.ncbi.nlm.nih.gov/nucleotide/KY616981.1?report=genbank&log$=nucltop&blast_rank=1&RID=DX8PTAHN01R) |
| LC6 |  | 9.00E-52 | 100.00% | [KY616975.1](https://www.ncbi.nlm.nih.gov/nucleotide/KY616975.1?report=genbank&log$=nucltop&blast_rank=1&RID=DX8T59VR014) |
| LC7 |  | 9.00E-52 | 100.00% | [KY616981.1](https://www.ncbi.nlm.nih.gov/nucleotide/KY616981.1?report=genbank&log$=nucltop&blast_rank=1&RID=DX8VVG6501R) |
| LC8 |  | 9.00E-52 | 100.00% | [KY616981.1](https://www.ncbi.nlm.nih.gov/nucleotide/KY616981.1?report=genbank&log$=nucltop&blast_rank=1&RID=91MKMVXP015) |
|  |  |  |  |  |
| EM1 | *Elephas maximus* | 9.00E-52 | 100.00% | [KC561811.1](https://www.ncbi.nlm.nih.gov/nucleotide/KC561811.1?report=genbank&log$=nucltop&blast_rank=1&RID=91NVEY2E015) |
| EM2 |  | 9.00E-52 | 100.00% | [KC561811.1](https://www.ncbi.nlm.nih.gov/nucleotide/KC561811.1?report=genbank&log$=nucltop&blast_rank=1&RID=91NT6STM014) |
| EM3 |  | 9.00E-52 | 100.00% | [KC561811.1](https://www.ncbi.nlm.nih.gov/nucleotide/KC561811.1?report=genbank&log$=nucltop&blast_rank=1&RID=91NT6STM014) |
| EM4 |  | 9.00E-52 | 100.00% | [KC561811.1](https://www.ncbi.nlm.nih.gov/nucleotide/KC561811.1?report=genbank&log$=nucltop&blast_rank=1&RID=91NT6STM014) |
| **Sample Name** | **Species** | **E value** | **% NS** | **Matched species (Accession No)** |
| EM5 | *Elephas maximus* | 9.00E-52 | 100.00% | [FJ753557.1](https://www.ncbi.nlm.nih.gov/nucleotide/FJ753557.1?report=genbank&log$=nucltop&blast_rank=2&RID=DWY0M1JF014) |
| EM6 |  | 9.00E-52 | 100.00% | [KC561811.1](https://www.ncbi.nlm.nih.gov/nucleotide/KC561811.1?report=genbank&log$=nucltop&blast_rank=1&RID=91P2MDNE014) |
| EM7 |  | 9.00E-52 | 100.00% | [KC561811.1](https://www.ncbi.nlm.nih.gov/nucleotide/KC561811.1?report=genbank&log$=nucltop&blast_rank=1&RID=91P7VUG8014) |
| EM8 |  | 9.00E-52 | 100.00% | [KC561811.1](https://www.ncbi.nlm.nih.gov/nucleotide/KC561811.1?report=genbank&log$=nucltop&blast_rank=1&RID=91PFJKEW015) |
| EM9 |  | 9.00E-52 | 100.00% | [KC561811.1](https://www.ncbi.nlm.nih.gov/nucleotide/KC561811.1?report=genbank&log$=nucltop&blast_rank=1&RID=91PMPVBH013) |
| EM10 |  | 9.00E-52 | 100.00% | [KC561811.1](https://www.ncbi.nlm.nih.gov/nucleotide/KC561811.1?report=genbank&log$=nucltop&blast_rank=1&RID=91PPVUUA013) |
| EM11 |  | 9.00E-52 | 100.00% | [KC561811.1](https://www.ncbi.nlm.nih.gov/nucleotide/KC561811.1?report=genbank&log$=nucltop&blast_rank=1&RID=91PSK046013) |
| EM12 |  | 9.00E-52 | 100.00% | [KC561811.1](https://www.ncbi.nlm.nih.gov/nucleotide/KC561811.1?report=genbank&log$=nucltop&blast_rank=1&RID=91PVUTRY013) |
| EM13 |  | 9.00E-52 | 100.00% | [FJ753557.1](https://www.ncbi.nlm.nih.gov/nucleotide/FJ753557.1?report=genbank&log$=nucltop&blast_rank=2&RID=DWY0M1JF014) |
| EM14 |  | 9.00E-52 | 100.00% | [FJ753557.1](https://www.ncbi.nlm.nih.gov/nucleotide/FJ753557.1?report=genbank&log$=nucltop&blast_rank=2&RID=DWY0M1JF014) |
| EM15 |  | 9.00E-52 | 100.00% | [KC561811.1](https://www.ncbi.nlm.nih.gov/nucleotide/KC561811.1?report=genbank&log$=nucltop&blast_rank=1&RID=91R4R9DV013) |
| EM16 |  | 9.00E-52 | 100.00% | [KC561811.1](https://www.ncbi.nlm.nih.gov/nucleotide/KC561811.1?report=genbank&log$=nucltop&blast_rank=1&RID=91R7ZMT4013) |
| EM17 |  | 9.00E-52 | 100.00% | [KC561811.1](https://www.ncbi.nlm.nih.gov/nucleotide/KC561811.1?report=genbank&log$=nucltop&blast_rank=1&RID=91R7ZMT4013) |
| EM18 |  | 9.00E-52 | 100.00% | [KC561811.1](https://www.ncbi.nlm.nih.gov/nucleotide/KC561811.1?report=genbank&log$=nucltop&blast_rank=1&RID=91R7ZMT4013) |
| EM19 |  | 9.00E-52 | 100.00% | [KC561811.1](https://www.ncbi.nlm.nih.gov/nucleotide/KC561811.1?report=genbank&log$=nucltop&blast_rank=1&RID=91R7ZMT4013) |
| EM20 |  | 9.00E-52 | 100.00% | [KC561811.1](https://www.ncbi.nlm.nih.gov/nucleotide/KC561811.1?report=genbank&log$=nucltop&blast_rank=1&RID=91R7ZMT4013) |
| EM21 |  | 9.00E-52 | 100.00% | [KC561811.1](https://www.ncbi.nlm.nih.gov/nucleotide/KC561811.1?report=genbank&log$=nucltop&blast_rank=1&RID=91R7ZMT4013) |
| EM22 |  | 9.00E-52 | 100.00% | [KC561811.1](https://www.ncbi.nlm.nih.gov/nucleotide/KC561811.1?report=genbank&log$=nucltop&blast_rank=1&RID=91R7ZMT4013) |
| EM23 |  | 9.00E-52 | 100.00% | [KC561811.1](https://www.ncbi.nlm.nih.gov/nucleotide/KC561811.1?report=genbank&log$=nucltop&blast_rank=1&RID=91R7ZMT4013) |
| EM24 |  | 9.00E-52 | 100.00% | [KC561811.1](https://www.ncbi.nlm.nih.gov/nucleotide/KC561811.1?report=genbank&log$=nucltop&blast_rank=1&RID=91R7ZMT4013) |
| EM25 |  | 9.00E-52 | 100.00% | [KC561811.1](https://www.ncbi.nlm.nih.gov/nucleotide/KC561811.1?report=genbank&log$=nucltop&blast_rank=1&RID=91R7ZMT4013) |
| EM26 |  | 9.00E-52 | 100.00% | [KC561811.1](https://www.ncbi.nlm.nih.gov/nucleotide/KC561811.1?report=genbank&log$=nucltop&blast_rank=1&RID=91R7ZMT4013) |
| EM27 |  | 9.00E-52 | 100.00% | [KC561811.1](https://www.ncbi.nlm.nih.gov/nucleotide/KC561811.1?report=genbank&log$=nucltop&blast_rank=1&RID=91R7ZMT4013) |
| EM28 |  | 9.00E-52 | 100.00% | [KC561811.1](https://www.ncbi.nlm.nih.gov/nucleotide/KC561811.1?report=genbank&log$=nucltop&blast_rank=1&RID=91R7ZMT4013) |
| EM29 |  | 9.00E-52 | 100.00% | [KC561811.1](https://www.ncbi.nlm.nih.gov/nucleotide/KC561811.1?report=genbank&log$=nucltop&blast_rank=1&RID=91R7ZMT4013) |
| EM30 |  | 9.00E-52 | 100.00% | [KC561811.1](https://www.ncbi.nlm.nih.gov/nucleotide/KC561811.1?report=genbank&log$=nucltop&blast_rank=1&RID=91R7ZMT4013) |
| EM31 |  | 9.00E-52 | 100.00% | [KC561811.1](https://www.ncbi.nlm.nih.gov/nucleotide/KC561811.1?report=genbank&log$=nucltop&blast_rank=1&RID=91R7ZMT4013) |
| EM32 |  | 9.00E-52 | 100.00% | [KC561811.1](https://www.ncbi.nlm.nih.gov/nucleotide/KC561811.1?report=genbank&log$=nucltop&blast_rank=1&RID=91R7ZMT4013) |
| EM33 |  | 9.00E-52 | 100.00% | [KC561811.1](https://www.ncbi.nlm.nih.gov/nucleotide/KC561811.1?report=genbank&log$=nucltop&blast_rank=1&RID=91R7ZMT4013) |
| EM34 |  | 9.00E-52 | 100.00% | [KC561811.1](https://www.ncbi.nlm.nih.gov/nucleotide/KC561811.1?report=genbank&log$=nucltop&blast_rank=1&RID=91R7ZMT4013) |
| EM35 |  | 9.00E-52 | 100.00% | [KC561811.1](https://www.ncbi.nlm.nih.gov/nucleotide/KC561811.1?report=genbank&log$=nucltop&blast_rank=1&RID=91R7ZMT4013) |
| EM36 |  | 9.00E-52 | 100.00% | [FJ753557.1](https://www.ncbi.nlm.nih.gov/nucleotide/FJ753557.1?report=genbank&log$=nucltop&blast_rank=2&RID=DWY0M1JF014) |
| EM37 |  | 9.00E-52 | 100.00% | [KC561811.1](https://www.ncbi.nlm.nih.gov/nucleotide/KC561811.1?report=genbank&log$=nucltop&blast_rank=1&RID=91R7ZMT4013) |
| EM38 |  | 9.00E-52 | 100.00% | [KC561811.1](https://www.ncbi.nlm.nih.gov/nucleotide/KC561811.1?report=genbank&log$=nucltop&blast_rank=1&RID=91R7ZMT4013) |
| EM39 |  | 9.00E-52 | 100.00% | [KC561811.1](https://www.ncbi.nlm.nih.gov/nucleotide/KC561811.1?report=genbank&log$=nucltop&blast_rank=1&RID=91R7ZMT4013) |
| EM40 |  | 9.00E-52 | 100.00% | [KC561811.1](https://www.ncbi.nlm.nih.gov/nucleotide/KC561811.1?report=genbank&log$=nucltop&blast_rank=1&RID=91R7ZMT4013) |
| EM41 |  | 9.00E-52 | 100.00% | [KC561811.1](https://www.ncbi.nlm.nih.gov/nucleotide/KC561811.1?report=genbank&log$=nucltop&blast_rank=1&RID=91R7ZMT4013) |
| EM42 |  | 9.00E-52 | 100.00% | [KC561811.1](https://www.ncbi.nlm.nih.gov/nucleotide/KC561811.1?report=genbank&log$=nucltop&blast_rank=1&RID=91R7ZMT4013) |
| EM43 |  | 9.00E-52 | 100.00% | [KC561811.1](https://www.ncbi.nlm.nih.gov/nucleotide/KC561811.1?report=genbank&log$=nucltop&blast_rank=1&RID=91R7ZMT4013) |
| **Sample Name** | **Species** | **E value** | **% NS** | **Matched species (Accession No)** |
| EM44 | *Elephas maximus* | 9.00E-52 | 100.00% | [KC561811.1](https://www.ncbi.nlm.nih.gov/nucleotide/KC561811.1?report=genbank&log$=nucltop&blast_rank=1&RID=91R7ZMT4013) |
| EM45 |  | 9.00E-52 | 100.00% | [KC561811.1](https://www.ncbi.nlm.nih.gov/nucleotide/KC561811.1?report=genbank&log$=nucltop&blast_rank=1&RID=91R7ZMT4013) |
| EM46 |  | 9.00E-52 | 100.00% | [KC561811.1](https://www.ncbi.nlm.nih.gov/nucleotide/KC561811.1?report=genbank&log$=nucltop&blast_rank=1&RID=91R7ZMT4013) |
| EM47 |  | 9.00E-52 | 100.00% | [KC561811.1](https://www.ncbi.nlm.nih.gov/nucleotide/KC561811.1?report=genbank&log$=nucltop&blast_rank=1&RID=91R7ZMT4013) |
| EM48 |  | 9.00E-52 | 100.00% | [KC561811.1](https://www.ncbi.nlm.nih.gov/nucleotide/KC561811.1?report=genbank&log$=nucltop&blast_rank=1&RID=91R7ZMT4013) |
| EM49 |  | 9.00E-52 | 100.00% | [KC561811.1](https://www.ncbi.nlm.nih.gov/nucleotide/KC561811.1?report=genbank&log$=nucltop&blast_rank=1&RID=9211SNCG014) |
| EM50 |  | 9.00E-52 | 100.00% | [FJ753557.1](https://www.ncbi.nlm.nih.gov/nucleotide/FJ753557.1?report=genbank&log$=nucltop&blast_rank=2&RID=DWY0M1JF014) |
| EM51 |  | 9.00E-52 | 100.00% | [FJ753557.1](https://www.ncbi.nlm.nih.gov/nucleotide/FJ753557.1?report=genbank&log$=nucltop&blast_rank=2&RID=DWY0M1JF014) |
| EM52 |  | 8.00E-58 | 99.22% | [KC561811.1](https://www.ncbi.nlm.nih.gov/nucleotide/KC561811.1?report=genbank&log$=nucltop&blast_rank=1&RID=928RXW5N015) |
| EM53 |  | 9.00E-52 | 100.00% | [KC561811.1](https://www.ncbi.nlm.nih.gov/nucleotide/KC561811.1?report=genbank&log$=nucltop&blast_rank=1&RID=91R7ZMT4013) |
| EM54 |  | 9.00E-52 | 100.00% | [KC561811.1](https://www.ncbi.nlm.nih.gov/nucleotide/KC561811.1?report=genbank&log$=nucltop&blast_rank=1&RID=91R7ZMT4013) |
| EM55 |  | 9.00E-52 | 100.00% | [KC561811.1](https://www.ncbi.nlm.nih.gov/nucleotide/KC561811.1?report=genbank&log$=nucltop&blast_rank=1&RID=921H7R2B015) |
| EM56 |  | 9.00E-52 | 100.00% | [KC561811.1](https://www.ncbi.nlm.nih.gov/nucleotide/KC561811.1?report=genbank&log$=nucltop&blast_rank=1&RID=91R7ZMT4013) |
| EM57 |  | 9.00E-52 | 100.00% | [KC561811.1](https://www.ncbi.nlm.nih.gov/nucleotide/KC561811.1?report=genbank&log$=nucltop&blast_rank=1&RID=91R7ZMT4013) |
| EM58 |  | 9.00E-52 | 100.00% | [KC561811.1](https://www.ncbi.nlm.nih.gov/nucleotide/KC561811.1?report=genbank&log$=nucltop&blast_rank=1&RID=91R7ZMT4013) |
| EM59 |  | 9.00E-52 | 100.00% | [KC561811.1](https://www.ncbi.nlm.nih.gov/nucleotide/KC561811.1?report=genbank&log$=nucltop&blast_rank=1&RID=91R7ZMT4013) |
| EM60 |  | 9.00E-52 | 100.00% | [KC561811.1](https://www.ncbi.nlm.nih.gov/nucleotide/KC561811.1?report=genbank&log$=nucltop&blast_rank=1&RID=91R7ZMT4013) |
| EM61 |  | 9.00E-52 | 100.00% | [KC561811.1](https://www.ncbi.nlm.nih.gov/nucleotide/KC561811.1?report=genbank&log$=nucltop&blast_rank=1&RID=91R7ZMT4013) |
|  |  |  |  |  |
| MP1 | *Mammuthus primigenius* | 9.00E-52 | 100.00% | [LC136999.1](https://www.ncbi.nlm.nih.gov/nucleotide/LC136999.1?report=genbank&log$=nucltop&blast_rank=1&RID=DX9054RF014) |
| MP2 |  | 9.00E-52 | 100.00% | [MH158736.1](https://www.ncbi.nlm.nih.gov/nucleotide/MH158736.1?report=genbank&log$=nucltop&blast_rank=1&RID=922FGHCW014) |
| MP3 |  | 9.00E-52 | 100.00% | [LC136999.1](https://www.ncbi.nlm.nih.gov/nucleotide/LC136999.1?report=genbank&log$=nucltop&blast_rank=1&RID=DX95UVPR014) |
| MP4 |  | 9.00E-52 | 100.00% | [LC136999.1](https://www.ncbi.nlm.nih.gov/nucleotide/LC136999.1?report=genbank&log$=nucltop&blast_rank=1&RID=DX95UVPR014) |
| MP5 |  | 9.00E-52 | 100.00% | [LC136999.1](https://www.ncbi.nlm.nih.gov/nucleotide/LC136999.1?report=genbank&log$=nucltop&blast_rank=1&RID=DX95UVPR014) |
| MP6 |  | 9.00E-52 | 100.00% | [MH158736.1](https://www.ncbi.nlm.nih.gov/nucleotide/MH158736.1?report=genbank&log$=nucltop&blast_rank=1&RID=923KC4Z1014) |
| MP7 |  | 9.00E-52 | 100.00% | [MH158736.1](https://www.ncbi.nlm.nih.gov/nucleotide/MH158736.1?report=genbank&log$=nucltop&blast_rank=1&RID=923KC4Z1014) |
| MP8 |  | 9.00E-52 | 100.00% | [LC136999.1](https://www.ncbi.nlm.nih.gov/nucleotide/LC136999.1?report=genbank&log$=nucltop&blast_rank=1&RID=DX9CPKKS015) |
| MP9 |  | 9.00E-52 | 100.00% | [MH158736.1](https://www.ncbi.nlm.nih.gov/nucleotide/MH158736.1?report=genbank&log$=nucltop&blast_rank=2&RID=DX9G6XS8014) |
| MP10 |  | 9.00E-52 | 100.00% | [MG334282.1](https://www.ncbi.nlm.nih.gov/nucleotide/MG334282.1?report=genbank&log$=nucltop&blast_rank=1&RID=923ZYTH0015) |
| MP11 |  | 9.00E-52 | 100.00% | [MH158736.1](https://www.ncbi.nlm.nih.gov/nucleotide/MH158736.1?report=genbank&log$=nucltop&blast_rank=1&RID=9242ZJ31015) |
| MP12 |  | 9.00E-52 | 100.00% | [MH158736.1](https://www.ncbi.nlm.nih.gov/nucleotide/MH158736.1?report=genbank&log$=nucltop&blast_rank=1&RID=9244VDBM015) |
| MP13 |  | 9.00E-52 | 100.00% | [MH158736.1](https://www.ncbi.nlm.nih.gov/nucleotide/MH158736.1?report=genbank&log$=nucltop&blast_rank=1&RID=9246P0ET014) |
| MP14 |  | 9.00E-52 | 100.00% | [MG334284.1](https://www.ncbi.nlm.nih.gov/nucleotide/MG334284.1?report=genbank&log$=nucltop&blast_rank=2&RID=9248N0CC015) |
| MP15 |  | 9.00E-52 | 100.00% | [LC136999.1](https://www.ncbi.nlm.nih.gov/nucleotide/LC136999.1?report=genbank&log$=nucltop&blast_rank=1&RID=DX9CPKKS015) |
| MP16 |  | 9.00E-52 | 100.00% | [MG334281.1](https://www.ncbi.nlm.nih.gov/nucleotide/MG334281.1?report=genbank&log$=nucltop&blast_rank=3&RID=924BD6K9014) |
| MP17 |  | 9.00E-52 | 100.00% | [MG334282.1](https://www.ncbi.nlm.nih.gov/nucleotide/MG334282.1?report=genbank&log$=nucltop&blast_rank=1&RID=DX9NF0CG014) |
| MP18 |  | 9.00E-52 | 100.00% | [LC136999.1](https://www.ncbi.nlm.nih.gov/nucleotide/LC136999.1?report=genbank&log$=nucltop&blast_rank=1&RID=DXBGS0DK014) |
| MP19 |  | 9.00E-52 | 100.00% | [LC136999.1](https://www.ncbi.nlm.nih.gov/nucleotide/LC136999.1?report=genbank&log$=nucltop&blast_rank=1&RID=DXBGS0DK014) |
| MP20 |  | 9.00E-52 | 100.00% | [LC136999.1](https://www.ncbi.nlm.nih.gov/nucleotide/LC136999.1?report=genbank&log$=nucltop&blast_rank=1&RID=DXBGS0DK014) |
| **Sample Name** | **Species** | **E value** | **% NS** | **Matched species (Accession No)** |
| MP21 | *Mammuthus primigenius* | 9.00E-52 | 100.00% | [LC136999.1](https://www.ncbi.nlm.nih.gov/nucleotide/LC136999.1?report=genbank&log$=nucltop&blast_rank=1&RID=DXBGS0DK014) |
| MP22 |  | 9.00E-52 | 100.00% | [LC136999.1](https://www.ncbi.nlm.nih.gov/nucleotide/LC136999.1?report=genbank&log$=nucltop&blast_rank=1&RID=DXBGS0DK014) |
| MP23 |  | 9.00E-52 | 100.00% | [LC136999.1](https://www.ncbi.nlm.nih.gov/nucleotide/LC136999.1?report=genbank&log$=nucltop&blast_rank=1&RID=DXBGS0DK014) |
| MP24 |  | 9.00E-52 | 100.00% | [LC136999.1](https://www.ncbi.nlm.nih.gov/nucleotide/LC136999.1?report=genbank&log$=nucltop&blast_rank=1&RID=DXBGS0DK014) |
| MP25 |  | 9.00E-52 | 100.00% | [LC136999.1](https://www.ncbi.nlm.nih.gov/nucleotide/LC136999.1?report=genbank&log$=nucltop&blast_rank=1&RID=DXBGS0DK014) |
| MP26 |  | 9.00E-52 | 100.00% | [LC136999.1](https://www.ncbi.nlm.nih.gov/nucleotide/LC136999.1?report=genbank&log$=nucltop&blast_rank=1&RID=DXBGS0DK014) |
| MP27 |  | 9.00E-52 | 100.00% | [LC136999.1](https://www.ncbi.nlm.nih.gov/nucleotide/LC136999.1?report=genbank&log$=nucltop&blast_rank=1&RID=DXBGS0DK014) |
| MP28 |  | 9.00E-52 | 100.00% | [LC136999.1](https://www.ncbi.nlm.nih.gov/nucleotide/LC136999.1?report=genbank&log$=nucltop&blast_rank=1&RID=DXBGS0DK014) |
| MP29 |  | 9.00E-52 | 100.00% | [LC136999.1](https://www.ncbi.nlm.nih.gov/nucleotide/LC136999.1?report=genbank&log$=nucltop&blast_rank=1&RID=DXBGS0DK014) |
| MP30 |  | 9.00E-52 | 100.00% | [LC136999.1](https://www.ncbi.nlm.nih.gov/nucleotide/LC136999.1?report=genbank&log$=nucltop&blast_rank=1&RID=DXBGS0DK014) |
| MP31 |  | 9.00E-52 | 100.00% | [LC136999.1](https://www.ncbi.nlm.nih.gov/nucleotide/LC136999.1?report=genbank&log$=nucltop&blast_rank=1&RID=DXBGS0DK014) |
| MP32 |  | 9.00E-52 | 100.00% | [LC136999.1](https://www.ncbi.nlm.nih.gov/nucleotide/LC136999.1?report=genbank&log$=nucltop&blast_rank=1&RID=DXBGS0DK014) |
| MP33 |  | 9.00E-52 | 100.00% | [LC136999.1](https://www.ncbi.nlm.nih.gov/nucleotide/LC136999.1?report=genbank&log$=nucltop&blast_rank=1&RID=DXBGS0DK014) |
| MP34 |  | 9.00E-52 | 100.00% | [LC136999.1](https://www.ncbi.nlm.nih.gov/nucleotide/LC136999.1?report=genbank&log$=nucltop&blast_rank=1&RID=DXBGS0DK014) |
| MP35 |  | 9.00E-52 | 100.00% | [LC136999.1](https://www.ncbi.nlm.nih.gov/nucleotide/LC136999.1?report=genbank&log$=nucltop&blast_rank=1&RID=DXBGS0DK014) |
| MP36 |  | 9.00E-52 | 100.00% | [LC136999.1](https://www.ncbi.nlm.nih.gov/nucleotide/LC136999.1?report=genbank&log$=nucltop&blast_rank=1&RID=DXBGS0DK014) |
| MP37 |  | 9.00E-52 | 100.00% | [MG334282.1](https://www.ncbi.nlm.nih.gov/nucleotide/MG334282.1?report=genbank&log$=nucltop&blast_rank=1&RID=DXG0JD7H015) |
| MP38 |  | 9.00E-52 | 100.00% | [MG334282.1](https://www.ncbi.nlm.nih.gov/nucleotide/MG334282.1?report=genbank&log$=nucltop&blast_rank=1&RID=DXG0JD7H015) |
| MP39 |  | 9.00E-52 | 100.00% | [LC136999.1](https://www.ncbi.nlm.nih.gov/nucleotide/LC136999.1?report=genbank&log$=nucltop&blast_rank=1&RID=DXBGS0DK014) |
| MP40 |  | 9.00E-52 | 100.00% | [LC136999.1](https://www.ncbi.nlm.nih.gov/nucleotide/LC136999.1?report=genbank&log$=nucltop&blast_rank=1&RID=DXBGS0DK014) |
| MP41 |  | 9.00E-52 | 100.00% | [MG334284.1](https://www.ncbi.nlm.nih.gov/nucleotide/MG334284.1?report=genbank&log$=nucltop&blast_rank=2&RID=926RJRWM014) |
| MP42 |  | 9.00E-52 | 100.00% | [MG334284.1](https://www.ncbi.nlm.nih.gov/nucleotide/MG334284.1?report=genbank&log$=nucltop&blast_rank=2&RID=926RJRWM014) |
| MP43 |  | 9.00E-52 | 100.00% | [MG334284.1](https://www.ncbi.nlm.nih.gov/nucleotide/MG334284.1?report=genbank&log$=nucltop&blast_rank=2&RID=926RJRWM014) |
| MP44 |  | 9.00E-52 | 100.00% | [MG334284.1](https://www.ncbi.nlm.nih.gov/nucleotide/MG334284.1?report=genbank&log$=nucltop&blast_rank=2&RID=926RJRWM014) |
| MP45 |  | 9.00E-52 | 100.00% | [LC136999.1](https://www.ncbi.nlm.nih.gov/nucleotide/LC136999.1?report=genbank&log$=nucltop&blast_rank=1&RID=DXBGS0DK014) |
| MP46 |  | 9.00E-52 | 100.00% | [LC136999.1](https://www.ncbi.nlm.nih.gov/nucleotide/LC136999.1?report=genbank&log$=nucltop&blast_rank=1&RID=DXBGS0DK014) |
| MP47 |  | 9.00E-52 | 100.00% | [MG334282.1](https://www.ncbi.nlm.nih.gov/nucleotide/MG334282.1?report=genbank&log$=nucltop&blast_rank=1&RID=DXGZUZ9G014) |
| MP48 |  | 9.00E-52 | 100.00% | [MG334282.1](https://www.ncbi.nlm.nih.gov/nucleotide/MG334282.1?report=genbank&log$=nucltop&blast_rank=1&RID=DXH43P3X014) |
| MP49 |  | 9.00E-52 | 100.00% | [LC136999.1](https://www.ncbi.nlm.nih.gov/nucleotide/LC136999.1?report=genbank&log$=nucltop&blast_rank=1&RID=DXBGS0DK014) |
| MP50 |  | 9.00E-52 | 100.00% | [LC136999.1](https://www.ncbi.nlm.nih.gov/nucleotide/LC136999.1?report=genbank&log$=nucltop&blast_rank=1&RID=DXBGS0DK014) |
| MP51 |  | 9.00E-52 | 100.00% | [LC136999.1](https://www.ncbi.nlm.nih.gov/nucleotide/LC136999.1?report=genbank&log$=nucltop&blast_rank=1&RID=DXBGS0DK014) |
| MP52 |  | 9.00E-52 | 100.00% | [LC136999.1](https://www.ncbi.nlm.nih.gov/nucleotide/LC136999.1?report=genbank&log$=nucltop&blast_rank=1&RID=DXBGS0DK014) |
| MP53 |  | 9.00E-52 | 100.00% | [LC136999.1](https://www.ncbi.nlm.nih.gov/nucleotide/LC136999.1?report=genbank&log$=nucltop&blast_rank=1&RID=DXBGS0DK014) |
| MP54 |  | 9.00E-52 | 100.00% | [LC136999.1](https://www.ncbi.nlm.nih.gov/nucleotide/LC136999.1?report=genbank&log$=nucltop&blast_rank=1&RID=DXBGS0DK014) |
| MP55 |  | 9.00E-52 | 100.00% | [LC136999.1](https://www.ncbi.nlm.nih.gov/nucleotide/LC136999.1?report=genbank&log$=nucltop&blast_rank=1&RID=DXBGS0DK014) |
| MP56 |  | 9.00E-52 | 100.00% | [LC136999.1](https://www.ncbi.nlm.nih.gov/nucleotide/LC136999.1?report=genbank&log$=nucltop&blast_rank=1&RID=DXBGS0DK014) |
| MP57 |  | 9.00E-52 | 100.00% | [LC136999.1](https://www.ncbi.nlm.nih.gov/nucleotide/LC136999.1?report=genbank&log$=nucltop&blast_rank=1&RID=DXBGS0DK014) |
| MP58 |  | 9.00E-52 | 100.00% | [LC136999.1](https://www.ncbi.nlm.nih.gov/nucleotide/LC136999.1?report=genbank&log$=nucltop&blast_rank=1&RID=DXBGS0DK014) |
| MP59 |  | 9.00E-52 | 100.00% | [LC136999.1](https://www.ncbi.nlm.nih.gov/nucleotide/LC136999.1?report=genbank&log$=nucltop&blast_rank=1&RID=DXBGS0DK014) |

**Table S3**. Complete cytochrome *b* sequences downloaded from GenBank and used in designing primers.

| **Species** | **Nomenclature** | **Accession number** | **Clade** | **Reference** |
| --- | --- | --- | --- | --- |
| African savanna elephant | *Loxodonta africana* | JQ438206 | F-West central | ^1^ |
|  | *Loxodonta africana* | JQ438366 | S-Southeast savanna | ^1^ |
|  | *Loxodonta africana* | JQ438457 | S-Southeast savanna | ^1^ |
|  | *Loxodonta africana* | JQ438588 | F-South-central | ^1^ |
|  | *Loxodonta africana* | JQ438594 | S-Savanna-wide | ^1^ |
|  | *Loxodonta africana* | JQ438674 | S-Southeast savanna | ^1^ |
|  | *Loxodonta africana* | JQ438713 | F-East-central | ^1^ |
|  | *Loxodonta africana* | JQ438746 | S-Northern-savanna | ^1^ |
|  |  |  |  |  |
| African Forest elephant | *Loxodonta cyclotis* | AY359269 | F-East-central | ^2^ |
|  | *Loxodonta cyclotis* | AY359279 | F-East-central | ^2^ |
|  | *Loxodonta cyclotis* | AY359271 | F-East-central | ^2^ |
|  | *Loxodonta cyclotis* | AY359270 | F-East-central | ^2^ |
|  | *Loxodonta cyclotis* | AY359272 | F-East-central | ^2^ |
|  | *Loxodonta cyclotis* | AY359268 | F-East-central | ^2^ |
|  | *Loxodonta cyclotis* | JQ438308 | F-North-central | ^1^ |
|  | *Loxodonta cyclotis* | JQ438501 | F-Western | ^1^ |
|  | *Loxodonta cyclotis* | JQ438512 | F-West-central | ^1^ |
|  | *Loxodonta cyclotis* | KJ557424.1 | F-West-central | ^3^ |
|  |  |  |  |  |
| Asian elephant | *Elephas maximus* | D50846 | α | ^4^ |
|  | *Elephas maximus* | AY769975 | α | ^5^ |
|  | *Elephas maximus* | AB002412 | β | ^4^ |
|  | *Elephas maximus* | D50844 | β | ^4^ |
|  |  |  |  |  |
| Woolly mammoth | *Mammuthus primigenus* | EU153446 | 1/DE | ^6^ |
|  | *Mammuthus primigenus* | EU153448 | 1/DE | ^6^ |
|  | *Mammuthus primigenus* | KX027500 | C | ^7^ |
|  | *Mammuthus primigenus* | KX027532 | C | ^7^ |
|  | *Mammuthus primigenus* | KX027531 | B1 | ^7^ |
|  | *Mammuthus primigenus* | MF579931 | B2 | ^8^ |
|  | *Mammuthus primigenus* | MF579934 | B2 | ^8^ |
|  | *Mammuthus primigenus* | EU153451 | 2/A | ^6^ |
|  | *Mammuthus primigenus* | EU153450 | 2/A | ^6^ |
| **Species** | **Nomenclature** | **Accession number** | **Clade** | **Reference** |
| Hippopotamus | *Hippopotamus amphibius* | U07565 | - | ^9^ |
|  | *Hippopotamus amphibius* | NC000889 | - | ^10^ |
| Domestic cow | *Bos taurus* | DQ186289 | - | ^11^ |
|  | *Bos taurus* | DQ186222 | - | ^11^ |
|  | *Bos taurus* | DQ186283 | - | ^11^ |
| White rhino | *Ceratotherium simum* | Y07726 | - | ^12^ |
|  | *Ceratotherium simum* | JF718874 | - | ^13^ |
|  | *Ceratotherium simum* | NC001808 | - | ^14^ |
| Human | *Homo sapiens* | NC_012920 | - | ^15^ |
| Killer whale | *Orcinus orca* | AF084060 | - | ^16^ |
|  | *Orcinus orca* | AF084061 | - | ^16^ |
| Narwhal | *Monodon monoceros* | X92532 | - | ^17^ |
|  | *Monodon monoceros* | U72038 | - | ^18^ |
|  | *Monodon monoceros* | AJ554062 | - | ^19^ |
| Desert warthog | *Phacochoerus aethiopicus* | AJ314551 | - | ^20^ |
|  | *Phacochoerus aethiopicus* | AJ314550 | - | ^20^ |
|  | *Phacochoerus aethiopicus* | AJ314549 | - | ^20^ |
| Sperm whale | *Physeter catodon* | KU891393 | - | ^21^ |
|  | *Physeter catodon* | KU891394 | - | ^21^ |
|  | *Physeter catodon* | KU891385 | - | ^21^ |
| Walrus | *Odobenus rosmarus* | GU174611 | - | ^22^ |
|  | *Odobenus rosmarus* | X82299 | - | ^23^ |

**References**

^1^Ishida, Y., Georgiadis, N. J., Hondo, T. & Roca, A. L. Triangulating the provenance of African elephants using mitochondrial DNA. Evolutionary Applications **6**, 253-265, doi:https://doi.org/110.1111/j.1752-4571.2012.00286.x (2013).

^2^Debruyne, R., Van Holt, A., Barriel, V. & Tassy, P. Status of the so-called African pygmy elephant (*Loxodonta pumilio* (Noack 1906)): phylogeny of cytochrome b and mitochondrial control region sequences. Comptes rendus biologies **326**, 687-697, doi:https://doi.org/10.1016/S1631-0691(03)00158-6 (2003).

^3^ Finch, T. M., Zhao, N., Korkin, D., Frederick, K. H. & Eggert, L. S. Evidence of Positive Selection in Mitochondrial Complexes I and V of the African Elephant. PLoS One **9**, e92587, doi:https://doi.org/10.1371/journal.pone.0092587 (2014).

^4^Noro, M., Masuda, R., Dubrovo, I. A., Yoshida, M. C. & Kato, M. Molecular phylogenetic inference of the woolly mammoth *Mammuthus primigenius*, based on complete sequences of mitochondrial cytochrome b and 12S ribosomal RNA genes. J. Mol. Evol. **46**, 314-326, doi: https://doi.org/10.1007/PL00006308 (1998).

^5^Lei, R., Brenneman, R. & Louis, E. Genetic diversity in the North American captive African elephant collection. J. Zool. **275**, 252-267, doi:https://doi.org/10.1111/j.1469-7998.2008.00437.x (2008).

^6^Gilbert, M. T. P. et al. Intraspecific phylogenetic analysis of Siberian woolly mammoths using complete mitochondrial genomes. Proceedings of the National Academy of Sciences **105**, 8327-8332, doi: https://doi.org/10.1073/pnas.0802315105 (2008).

^7^Enk, J. et al. *Mammuthus* population dynamics in late Pleistocene North America: divergence, phylogeography, and introgression. Frontiers in Ecology and Evolution **4**, 42, doi:https://doi.org/10.3389/fevo.2016.00042 (2016).

^8^ Yates, J. A. F. et al. Central European Woolly Mammoth Population Dynamics: Insights from Late Pleistocene Mitochondrial Genomes. Sci. Rep. **7**, 17714, doi:https://doi.org/10.1038/s41598-017-17723-1 (2017).

^9^Irwin, D. M. & Árnason, Ú. Cytochromeb gene of marine mammals: Phylogeny and evolution. Journal of Mammalian Evolution **2**, 37-55, doi: https://doi.org/10.1007/BF01464349 (1994).

^10^Ursing, B. M. & Arnason, U. Analyses of mitochondrial genomes strongly support a hippopotamus-whale clade. Proc. R. Soc. Lond. B. Biol. Sci. **265**, 2251-2255, doi: https://doi.org/10.1098/rspb.1998.0567 (1998).

^11^Cai, X. et al. mtDNA diversity and genetic lineages of eighteen cattle breeds from *Bos taurus* and *Bos indicus* in China. Genetica **131**, 175-183, doi:https://doi.org/10.1007/s10709-006-9129-y (2007).

^12^ Nilsson, M. A., Gullberg, A., Spotorno, A. E., Arnason, U. & Janke, A. Radiation of extant marsupials after the K/T boundary: evidence from complete mitochondrial genomes. J. Mol. Evol. **57**, S3-S12, doi:https://doi.org/10.1007/s00239-003-0001-8 (2003).

^13^ Steiner, C. C. & Ryder, O. A. Molecular phylogeny and evolution of the Perissodactyla. Zoological Journal of the Linnean Society **163**, 1289-1303, doi:https://doi.org/10.1111/j.1096-3642.2011.00752.x (2011).

^14^Xu, X. & Arnason, U. The complete mitochondrial DNA sequence of the white rhinoceros, *Ceratotherium simum*, and comparison with the mtDNA sequence of the Indian rhinoceros, *Rhinoceros unicornis*. Mol. Phylogenet. Evol. **7**, 189-194, doi:https://doi.org/10.1006/mpev.1996.0385 (1997).

^15^Andrews, R. M. et al. Reanalysis and revision of the Cambridge reference sequence for human mitochondrial DNA. Nat. Genet. **23**, 147, doi:https://doi.org/10.1038/13779 (1999).

^16^ LeDuc, R. G., Perrin, W. F. & Dizon, A. E. Phylogenetic relationships among the delphinid cetaceans based on full cytochrome b sequences. Mar. Mamm. Sci. **15**, 619-648, doi: https://doi.org/10.1111/j.1748-7692.1999.tb00833.x (1999).

^17^Árnason, Ú. & Gullberg, A. Cytochrome b nucleotide sequences and the identification of five primary lineages of extant cetaceans. Mol. Biol. Evol. 13, 407-417, doi:https://doi.org/10.1093/oxfordjournals.molbev.a025599 (1996).

^18^Árnason, Ú. & Gullberg, A. Cytochrome b nucleotide sequences and the identification of five primary lineages of extant cetaceans. Mol. Biol. Evol. **13**, 407-417, doi:https://doi.org/10.1093/oxfordjournals.molbev.a025599 (1996).

^19^Arnason, U., Gullberg, A. & Janke, A. Mitogenomic analyses provide new insights into cetacean origin and evolution. Gene **333**, 27-34, doi:https://doi.org/10.1016/j.gene.2004.02.010 (2004).

^20^ Randi, E., Lucchini, V. & Aman, R. Evidence of two genetically deeply divergent species of warthog, *Phacochoerus africanus* *and P. aethiopicus* (Artiodactyla: Suiformes) in East Africa. Mammalian Biology-Zeitschrift für Säugetierkunde **67**, 91-96, doi:https://doi.org/10.1078/1616-5047-00013 (2002).

^21^ Morin, P. A. et al. Demography or selection on linked cultural traits or genes? Investigating the driver of low mtDNA diversity in the sperm whale using complementary mitochondrial and nuclear genome analyses. Mol. Ecol. **27**, 2604-2619, doi:https://doi.org/10.1111/mec.14698 (2018).

^22^ Fulton, T. L. & Strobeck, C. Multiple fossil calibrations, nuclear loci and mitochondrial genomes provide new insight into biogeography and divergence timing for true seals (Phocidae, Pinnipedia). Journal of Biogeography **37**, 814-829, doi:https://doi.org/10.1111/j.1365-2699.2010.02271.x (2010).

^23^Árnason, Ú., Bodin, K., Gullberg, A., Ledje, C. & Suzette, M. A molecular view of pinniped relationships with particular emphasis on the true seals. J. Mol. Evol. **40**, 78-85, doi:https://doi.org/10.1007/BF00166598 (1995).

**
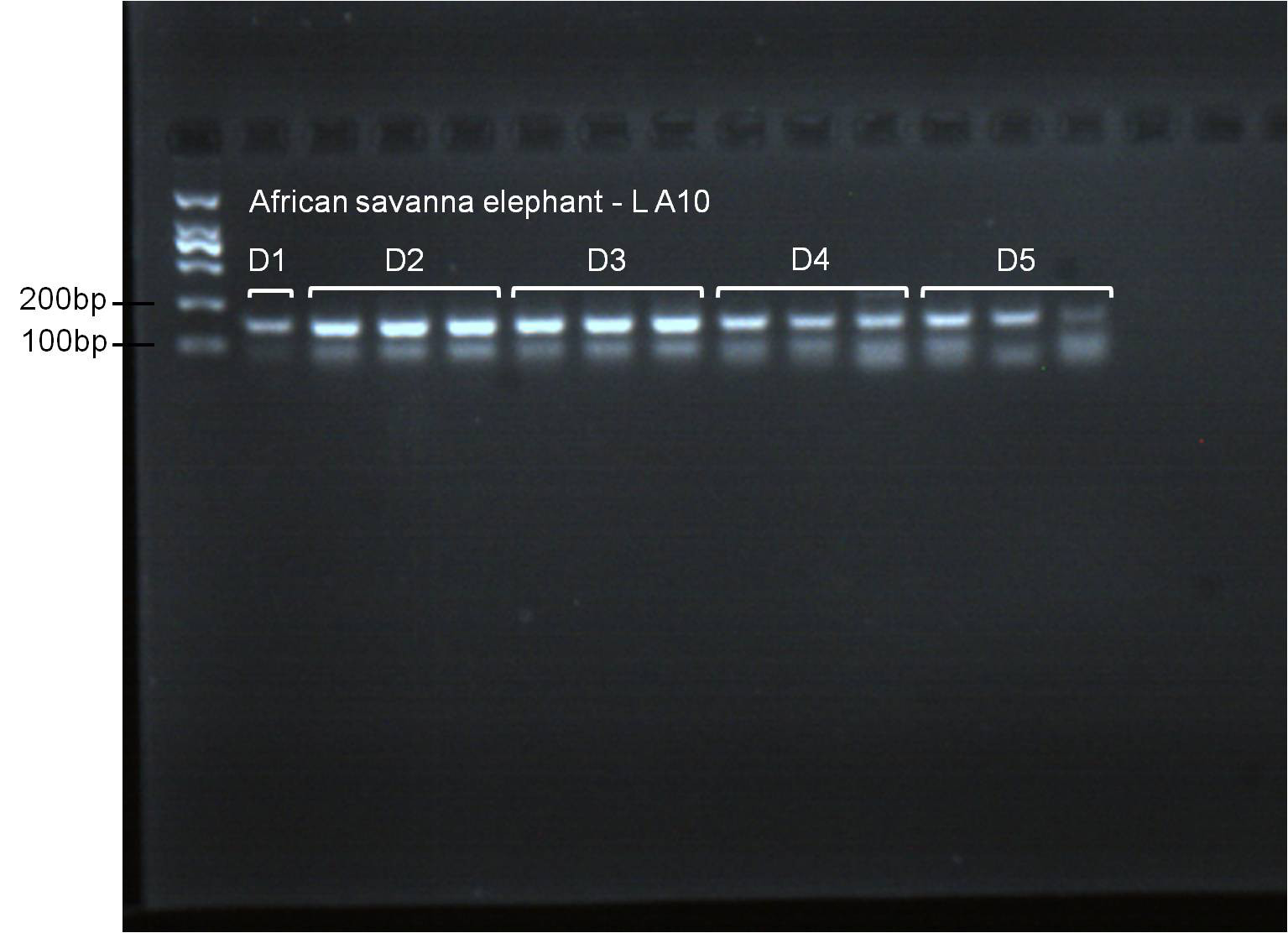

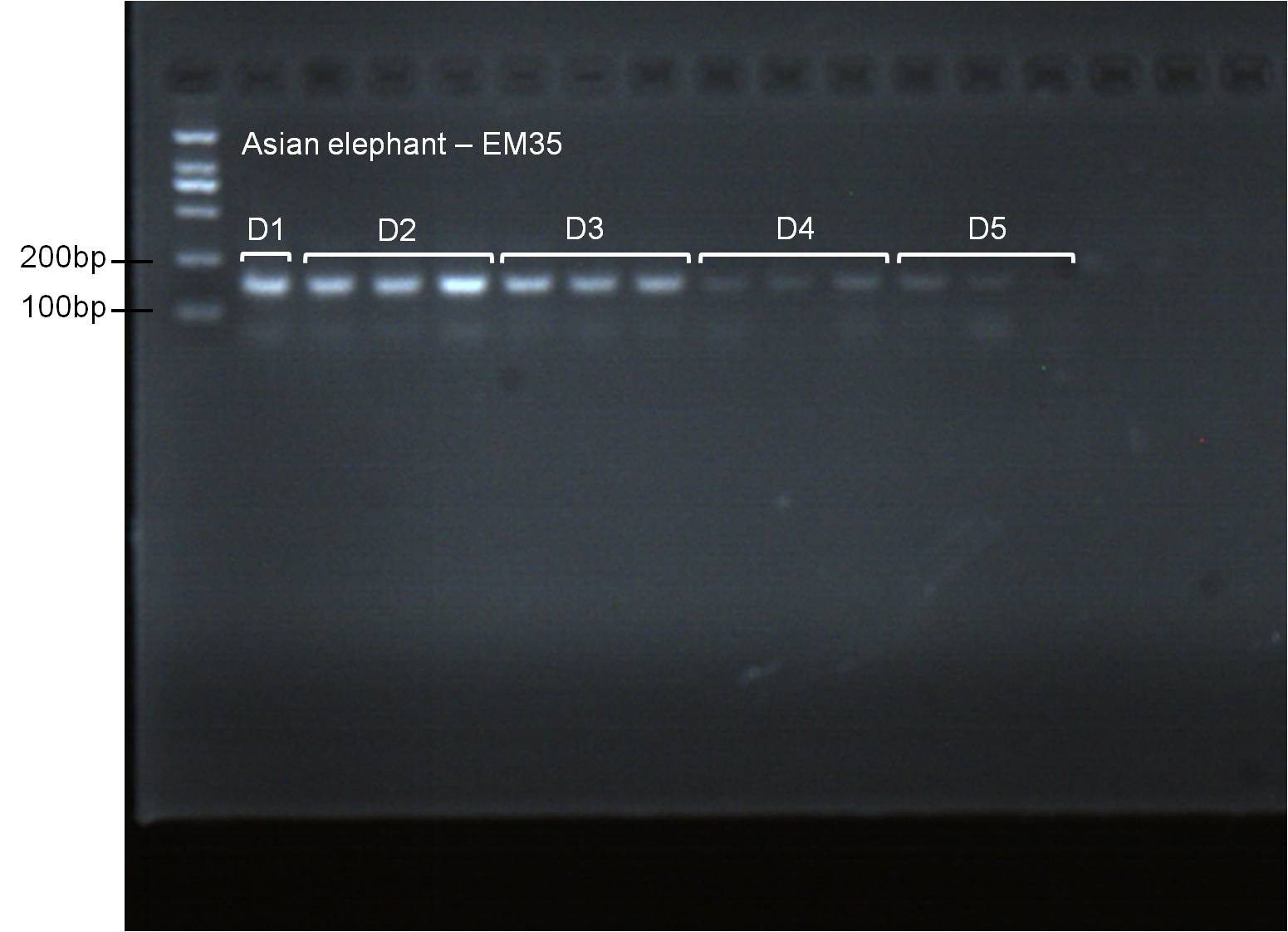
Figure S1.**


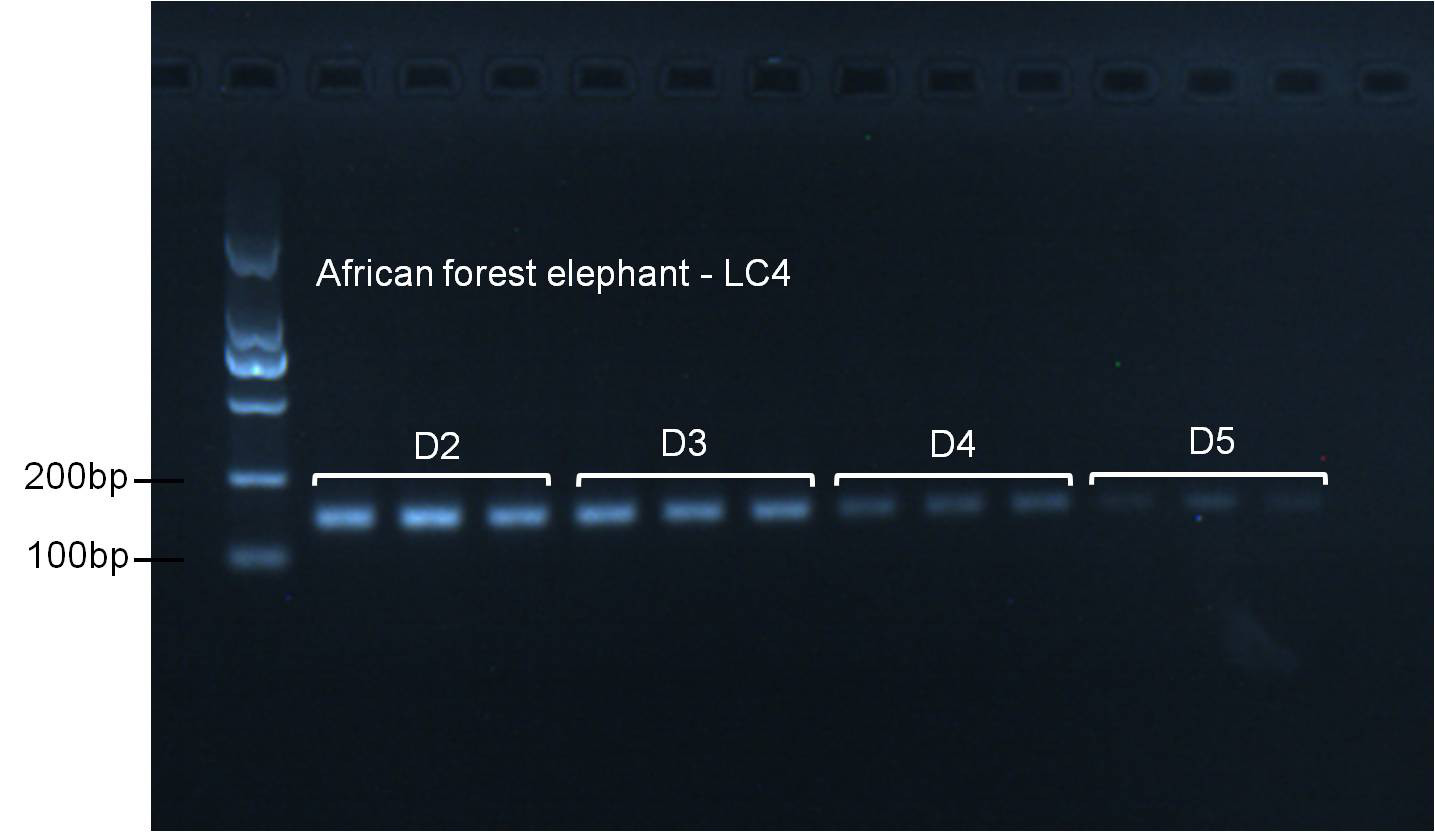

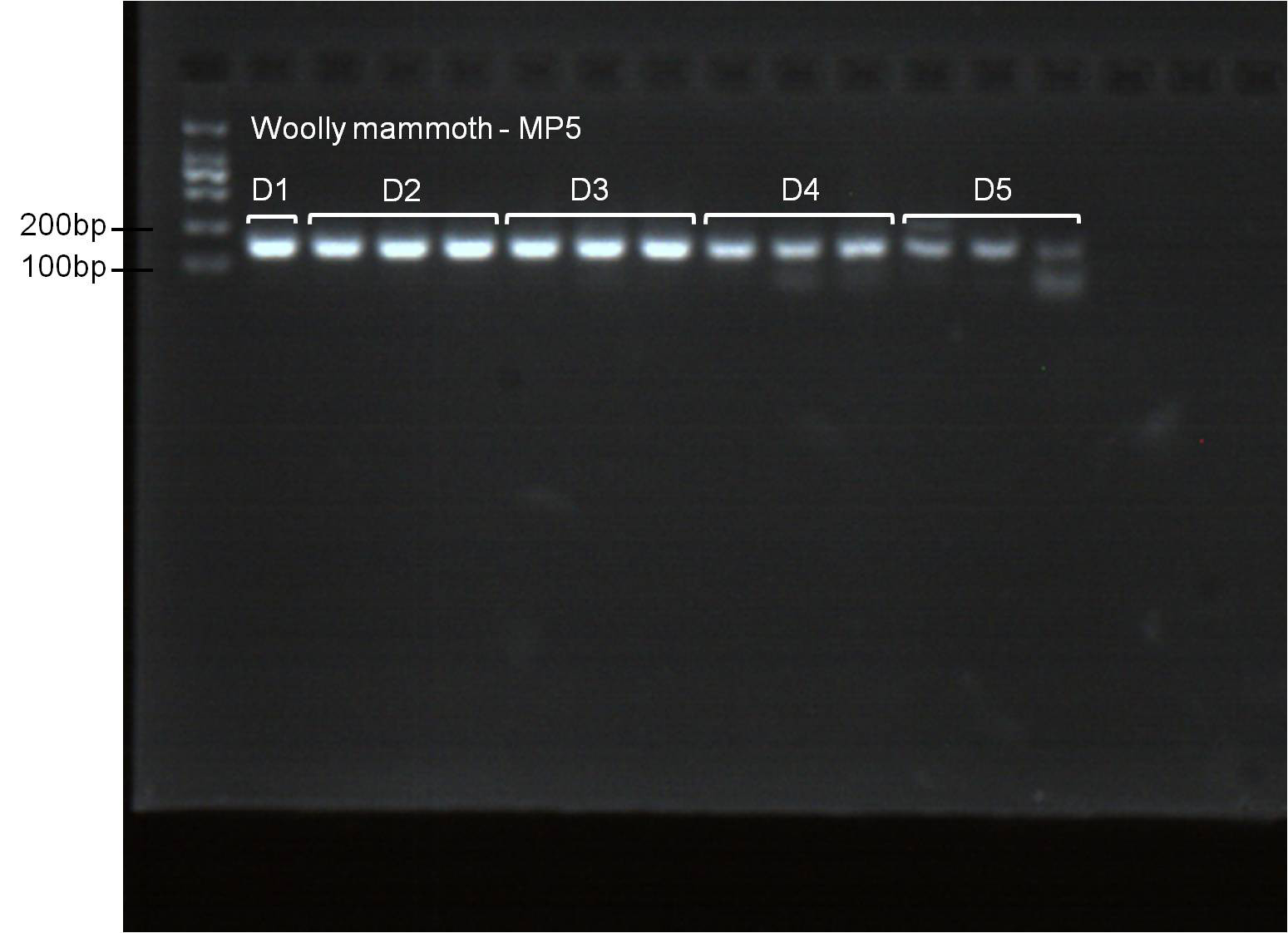

Supplement: Supplementary file 1 — Supplementary FigureS1, TableS1, TableS2, TableS3 [file 41598_2019_55094_MOESM1_ESM.docx]
